# Development of a Physiologically Based Model to Describe the Pharmacokinetics of Methylphenidate in Juvenile and Adult Humans and Nonhuman Primates

**DOI:** 10.1371/journal.pone.0106101

**Published:** 2014-09-03

**Authors:** Xiaoxia Yang, Suzanne M. Morris, Jeffery M. Gearhart, Christopher D. Ruark, Merle G. Paule, William Slikker, Donald R. Mattison, Benedetto Vitiello, Nathan C. Twaddle, Daniel R. Doerge, John F. Young, Jeffrey W. Fisher

**Affiliations:** 1 National Center for Toxicological Research, U.S. Food and Drug Administration, Jefferson, Arkansas, United States of America; 2 The Henry M. Jackson Foundation for the Advancement of Military Medicine, Wright-Patterson Air Force Base, Ohio, United States of America; 3 Risk Sciences International, Ottawa, Ontario, Canada; 4 University of Ottawa, Ottawa, Ontario, Canada; 5 National Institute of Mental Health, Bethesda, Maryland, United States of America; University of Kentucky, United States of America

## Abstract

The widespread usage of methylphenidate (MPH) in the pediatric population has received considerable attention due to its potential effect on child development. For the first time a physiologically based pharmacokinetic (PBPK) model has been developed in juvenile and adult humans and nonhuman primates to quantitatively evaluate species- and age-dependent enantiomer specific pharmacokinetics of MPH and its primary metabolite ritalinic acid. The PBPK model was first calibrated in adult humans using *in vitro* enzyme kinetic data of MPH enantiomers, together with plasma and urine pharmacokinetic data with MPH in adult humans. Metabolism of MPH in the small intestine was assumed to account for the low oral bioavailability of MPH. Due to lack of information, model development for children and juvenile and adult nonhuman primates primarily relied on intra- and interspecies extrapolation using allometric scaling. The juvenile monkeys appear to metabolize MPH more rapidly than adult monkeys and humans, both adults and children. Model prediction performance is comparable between juvenile monkeys and children, with average root mean squared error values of 4.1 and 2.1, providing scientific basis for interspecies extrapolation of toxicity findings. Model estimated human equivalent doses in children that achieve similar internal dose metrics to those associated with pubertal delays in juvenile monkeys were found to be close to the therapeutic doses of MPH used in pediatric patients. This computational analysis suggests that continued pharmacovigilance assessment is prudent for the safe use of MPH.

## Introduction

Attention deficit hyperactivity disorder (ADHD) is one of the most common childhood disorders and its frequent persistence into adulthood has been increasingly recognized [1]. According to a recent survey, the number of children in the U.S. diagnosed with ADHD continues to increase. Nearly 2 million additional U.S. children/adolescents aged 4 to 17 years were diagnosed with ADHD in 2011, compared to 2003 [2]. The point prevalence of ADHD is estimated to be 5–10% in children and about 3% in adults [1]. Methylphenidate (MPH), a blocker of the monoamine transporter that inhibits reuptake of dopamine and norepinephrine, remains a mainstay of treatment for ADHD [3]. Most MPH formulations contain a racemic mixture (1∶1) of the threo pair of MPH isomers (*d, l*-threo MPH), which is more potent pharmacologically than its corresponding erythro pair [4]–[6]. In addition, the *d-threo-*MPH (*d*-MPH) enantiomer exhibits a greater pharmacological potency than the *l*-enantiomer, and there is no evidence of interconversion between these two enantiomers [7]–[10]. Starting in 1960s, conventional, immediate-release MPH became the primary stimulant used to treat ADHD symptoms. Due to its short-term action (typically only lasting for 4 hours), IR MPH is typically given two to three times a day to cover normal school and after-school hours [11]. However, under such a dosing schedule, children may experience inattention during the trough in MPH levels, e.g. during late morning classes. Other problems associated with multiple dose regimens are compliance, confidentiality, and drug security issues at school. Given the dosing limitations of immediate-release MPH, several extended-release MPH formulations with longer effective durations of action have been introduced into the market [3], [12], [13].

**Figure 1:**
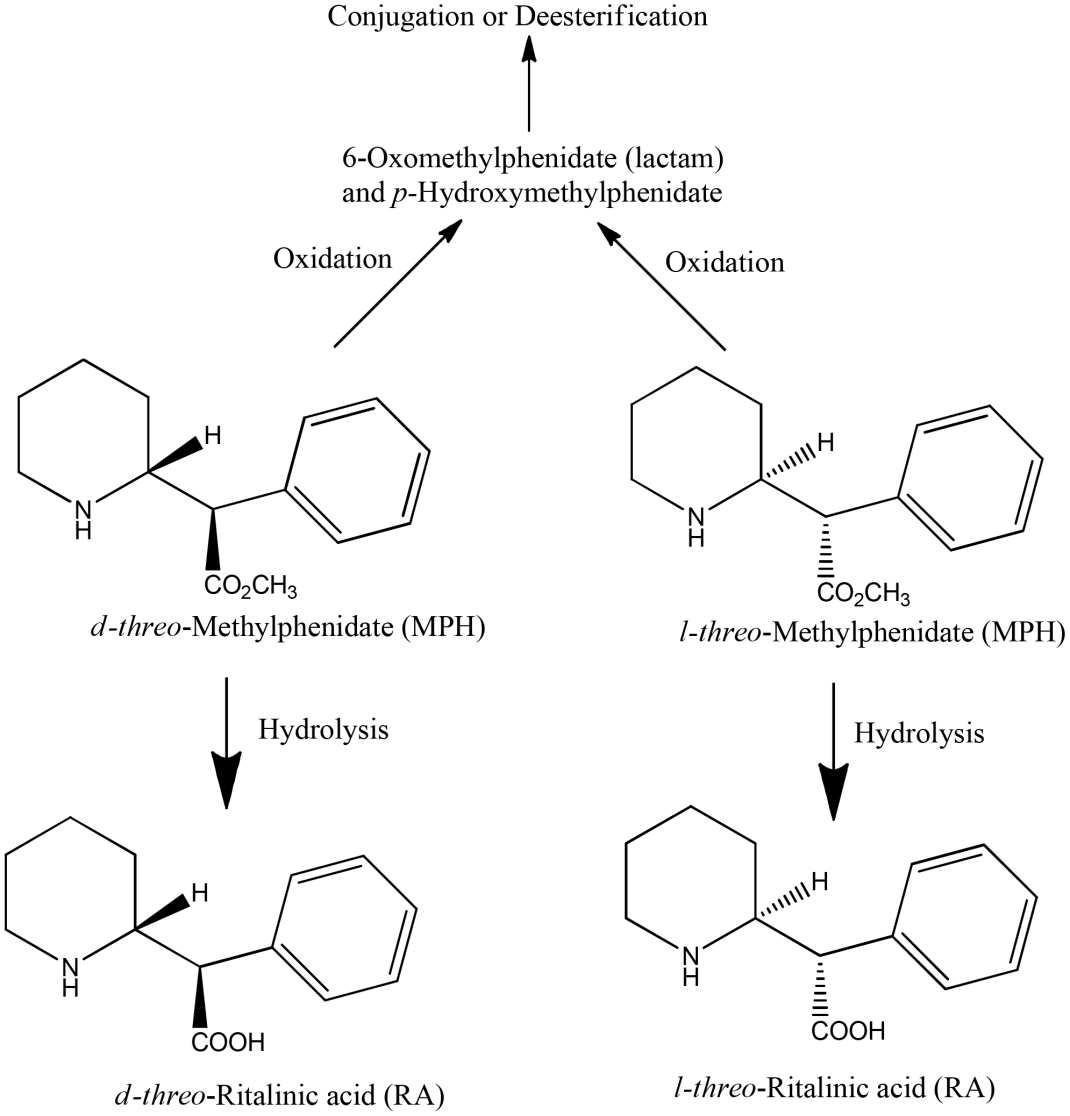
Metabolic pathways of methylphenidate in humans.

In humans, MPH is metabolized predominantly by hydrolysis (de-esterification) to the pharmacologically inactive ritalinic acid (RA), with pronounced enantioselectivity in favor of the *l*-enantiomer [4], [10], [13]–[15] (Figure 1). Human carboxylesterase (CES) 1A1 has been shown to be the major enzyme responsible for the stereoselective hydrolysis of MPH [16]. In addition, other minor metabolites produced through oxidation and subsequent conjugation or hydrolysis, including the pharmacologically active metabolite para-hydroxymethylphenidate [17], have also been identified in humans [18]. Extensive first-pass metabolism of total MPH (*d*-and *l*-MPH) results in low absolute oral bioavailability of the racemic drug. In healthy adult humans, only 22±8% and 5±3% of the *d*- and *l*-MPH, respectively, reach the systemic circulation [4]. In children diagnosed with hyperactivity, the systemic bioavailability of total MPH ranges from 11 to 52%, with an average of 31±16% [19]. The majority of MPH administered orally or by intravenous (iv) injection is excreted in urine, accounting for 80% [18] and 78–97% [20] of the administered dose within 48 h and 96 h, respectively. Only 3% of the administered MPH dose is recovered in feces over a 48 h period [18]. The major metabolite of MPH identified in urine is the hydrolytic metabolite RA, accounting for 80% of the total urinary excretion, following both oral and iv administration, while unmetabolized MPH accounted for less than 1% [13], [18].

The metabolism and disposition of MPH has been investigated in a variety of laboratory animals including rats, mice, and dogs [18], [21]. In contrast to humans, both microsomal oxidation and hydrolysis are important metabolic pathways for rats, mice, and dogs [18], [21]. In monkeys, RA (hydrolysis) has been shown to be a major metabolite of MPH [22], [23] and the oral bioavailability for total MPH was reported to be 22% in young monkeys [24].

Some health concerns exist for children and adults who are treated chronically with MPH. In a pediatric study to evaluate diurnal changes in salivary hormones of children taking psychotropic medications, those taking MPH tablets exhibited diminished diurnal rhythms of testosterone, while children taking extended-release MPH tablets had significantly higher testosterone levels [25]. In MPH toxicity studies in monkeys, juvenile male rhesus monkeys exhibited transient delays in puberty, lower serum testosterone levels, impaired testicular descent, and reduced testicular volume [26]. In another study, increases in blood testosterone levels were observed in peri-adolescent male rhesus monkeys [27].

To extrapolate the MPH toxicity findings reported in juvenile monkeys to children, a physiologically based pharmacokinetic (PBPK) model was constructed for MPH and its major metabolite RA for the first time. The model structure accounted for both the *d-* and *l-* enantiomers of MPH and RA in adult and young humans and non-human primates. The MPH PBPK model provided a computational methodology to evaluate and compare the pharmacokinetics of pharmacological doses of MPH in children with MPH doses used in the toxicity studies with juvenile rhesus monkeys. The metabolism and pharmacokinetics of MPH in young and adult humans have been evaluated for both immediate-release and extended-release MPH formulations. For juvenile and adult rhesus monkeys experiments conducted at the National Center for Toxicological Research (NCTR) in Jefferson, AR, only an immediate-release MPH formulation was used. Hence, to allow for cross-species comparison and extrapolation of MPH internal doses, only data obtained after the administration of immediate-release MPH are considered in the current manuscript.

## Materials and Methods

### Ethics Statement

All animal procedures were approved by the NCTR Institutional Animal Care and Use Committee.

### Key pharmacokinetic studies in humans

Given that *d-*MPH and *l-*MPH exhibit distinct pharmacokinetic profiles [4], [10], [14], [15] and pharmacological activities [7]–[10], simultaneous PBPK model predictions of both enantiomers are clinically relevant. Therefore, therapeutic drug monitoring studies utilizing enantiospecific assays were preferentially selected for human model development. In addition, pharmacokinetic studies with parallel measurements of MPH and its major metabolite RA concentrations were also considered important for tracking the mass balance of MPH and the fraction of MPH metabolized by the hydrolytic pathway. Pharmacokinetic data sets used for model calibration and evaluation for healthy adult humans and children with ADHD are briefly summarized below (Table S1 and Table S2). MPH used in these studies is assumed to consist of a 1∶1 racemic mixture of *d-* and *l-*enantiomers [4]–[6], [13], unless the use of *d-*MPH is indicated. In addition, unless specified otherwise, MPH and RA concentrations mentioned hereinafter refer to total (*d-* plus *l-*) MPH and total (*d*- plus *l-*) RA concentrations.

Pharmacokinetic data sets used to calibrate the adult human model were from iv and oral dosing studies [4], [24], [28]–[33]. For iv dosing, the first data set used to calibrate the model was time course of plasma *d-* and *l-*MPH concentrations following a single iv dose of 10 mg MPH in healthy adult men (n = 13) [4]. The second iv study used to calibrate the model was urinary excretion time course data for *d*- and *l*-RA in healthy adult men administered a single iv dose of 10 mg MPH (n = 9) [28]. For oral dosing, a total of six data sets were used for model calibration [24], [29]–[33], of which, three data sets provided time courses of plasma *d*- and *l*-MPH concentrations in healthy adults following a single oral dose of MPH at 0.3 mg/kg (n = 24) [29], 0.3 mg/kg (n = 19) [30], and 40 mg (n = 21) [31]; the other three studies reported time courses of plasma MPH and RA concentrations in healthy adult men following a single oral dose of MPH at 20 mg (n = 5) [32], 20 mg (n = 8) [33], and 0.15 and 0.3 mg/kg (n = 10) [24].

Pharmacokinetic data sets used to evaluate the adult human model were oral dosing studies [20], [28], [34]–[37]. The first kinetic studies used for testing the model were time courses of plasma d-MPH concentrations in healthy adults following a single oral dose of 50 or 90 mg MPH (n = 49) [34] and repeated oral doses of 30 mg MPH (n = 28) [35]. The second kinetic studies used for model evaluation were time courses of plasma MPH concentrations in healthy adults given a single oral dose of 20 mg MPH (n = 20) [36] and repeated oral doses of 5 mg MPH (n = 35), for which plasma RA concentrations were also determined [37]. The third kinetic studies used for model evaluation were urinary excretion time courses in healthy adult men for d- and l-RA following a single oral dose of 40 mg MPH (n = 9) [28] and for RA after a single oral dose of 20 mg MPH (n = 3) [20]. Additional plasma pharmacokinetic data sets in healthy adults administered either a single oral dose or repeated oral doses of MPH or d-MPH were also used for adult human model evaluation [38]–[43] (Text S1).

For children, one study reported serum MPH kinetics in boys administered 10–20 mg MPH intravenously (n = 6) [44] and another study reported serum peak RA levels in boys administered 10–15 mg MPH intravenously (n = 5) [19]. However, attempts to use these data sets for pediatric model development were not successful. Systemic clearance of MPH for children in the study of [44] is dramatically different from adult humans [4]. Hepatic metabolic constants describing MPH hydrolysis by human carboxylesterase 1 (hCES1) (see below for model description) need to be increased to approximately 50 fold of adult values to capture the rapid clearance of MPH observed for these children [44] while maintaining the appropriate estimation of serum RA levels [19]. Accordingly, the resultant scaled hepatic hydrolysis rate (µg/h) in children is 22–36 fold of adult human values. This is inconsistent with the finding that children show similar hepatic expression and activity of hCES1 enzyme compared to adult humans [45]. Further, plasma concentrations of MPH enantiomers following oral administration of 10 mg MPH in children [7], [46]–[51] are under estimated to a great extent using these large hepatic metabolic constants derived from these two studies [19], [44], even if assuming a rapid oral uptake and no gut metabolism. Eventually these two studies were excluded from data sets used for model development and evaluation because of their inconsistency with respect to several other MPH pharmacokinetic data sets in children [7], [46]–[51]. Hence, the pediatric MPH model was developed using MPH pharmacokinetic data sets following oral dosing in children [7], [19], [46]: of which, two studies provided plasma concentration time courses of d- and l-MPH following a single oral dose of 10 mg MPH in 5 boys with attention deficit disorder (ADD) [46] and 9 boys with ADHD [7]; and in another study, Chan et al. [19] reported peak serum RA levels in boys administered 10–15 mg MPH orally (n = 5).

Several additional pharmacokinetic data sets in children orally administered MPH were used for model evaluation [47]–[51]. The first data sets used for testing the model were plasma *d-*MPH concentration time courses in 14 preschool (4–5 years) and 9 school-aged (6–8 years) children with ADHD administered a single oral dose of 2.5–10 mg MPH [49] and in 31 boys with ADHD given a single oral dose of 5–20 mg MPH [50]. The second data sets used for model evaluation were plasma MPH concentration time courses in boys with ADD administered a single oral dose of MPH at 0.34 and 0.65 mg/kg (n = 14) [47], and in children with ADHD given repeated oral doses of MPH at 5–15 mg (normalized to a 5 mg dose, n = 14) [48] and 10–40 mg (normalized to a 20 mg dose, n = 14) [51].

### Key pharmacokinetic studies in nonhuman primates

The monkey MPH pharmacokinetic study reported by Wargin et al. [24] was used for model calibration. In this study, 5 young monkeys, aged 2.5 years, were dosed intravenously with 3 mg/kg of MPH and blood samples were collected over 9 hours [24]. Accordingly, MPH used for monkey studies is assumed to consist of a 1∶1 racemic mixture of *d-* and *l-*enantiomers [4]–[6], [13] as well.

Unreported pharmacokinetic data collected from a chronic MPH toxicity study at NCTR with juvenile rhesus monkeys [22], [23], [26] were also used to calibrate the monkey MPH PBPK model. The experimental design is briefly described below. In a preliminary study conducted to determine the most appropriate vehicle for MPH, 4 adult female rhesus monkeys (6.5–9.8 kg) were dosed with 0.3 mg/kg of MPH (USP grade, Mallinckrodt, St. Louis, MO) by oral bolus gavage (solution in Prang, Bio-Serv, Frenchtown, NJ) and via iv administration. Blood samples were collected at 13 time points after iv dosing and 9 time points after oral dosing over a 24 h period and plasma levels of both MPH and RA were determined by HPLC-MS/MS [52].

Based on plasma level data obtained from these studies in adult monkeys, a chronic toxicity study was performed with MPH and juvenile male rhesus monkeys [22], [23], [26]. Twenty male rhesus monkeys, approximately 2.5 years old at the beginning of the experiment (an age approximately equivalent to 7.5 year old boys, estimated based on maximum life-span of 122 and 40 years in humans and rhesus monkeys [53], [54]), were treated orally with MPH. The details of the study design and toxicity findings have been published in Morris et al. [23] and Mattison et al. [26]. Each lot of MPH (USP grade, Mallinckrodt, St. Louis, MO) was examined for purity prior to use in the study. All lots were determined to be structurally consistent with the NIST standard for MPH, with purity ≥99.0%. MPH was dissolved in Prang (Bio-Serv, Frenchtown, NJ), an oral rehydration solution commonly used as a vehicle in non-human primate experiments. Test article preparation occurred weekly and each dose preparation was analyzed by HPLC-MS/MS [52] for dose accuracy. Only dose preparations that were within ±10% of the target dose were used.

The test subjects were dosed twice a day (with a 4 hour interval), 5 days a week (Monday to Friday) via an oral dosing syringe. Both low dose (0.15 mg/kg of MPH, n = 10) and high doses (1.5 mg/kg of MPH, n = 10) were increased to final doses of 2.5 and 12.5 mg/kg [26]. These dose adjustments were required to achieve clinically relevant pediatric blood concentrations of MPH (2–10 ng/mL) [26], [55]. During the chronic MPH toxicity study, blood samples were collected after administration of MPH on a quarterly basis for about a 1-year period. On the days of blood collection (Monday to Thursday), the monkeys only received the first (morning) dose of MPH and blood samples were collected at eight time points from pre-dose to 24 h post-dose: samples were collected from 1–4 monkeys per time point. The monkeys underwent preliminary training for blood collection and were not anesthetized during the pharmacokinetic experiments. Plasma MPH and RA concentrations were determined for each monkey using an HPLC-MS/MS method [52]. Measurements of plasma MPH concentrations pre-dose and at 24 h post-dose were excluded due to quantitation limitations. Kinetic profiles of MPH and RA for each individual monkey were followed on the same day of the week when quarterly blood sampling occurred over the 1-year period.

Another nonhuman primate toxicity study with MPH [27], and limited plasma measurements of MPH, were used for evaluation of the monkey model. In this study, 8 male rhesus monkeys from Johns Hopkins University, approximately 3–4 years old, weighing 3.1–10.2 kg, were orally dosed with MPH in Tang solution via a 15-min self-dosing procedure, twice a day (at 0900 and 1200 hours), 7 days a week [27]. The average consumed MPH doses were 10.7 (8.89–13.1) and 16.5 (15.5–18.7) mg/kg, with the target intake determined to be 12–16 mg/kg, which produced the therapeutic blood levels of 15–25 ng/ml [27]. Blood sampling occurred periodically at 1000 and 1300 hours, and MPH plasma concentrations were quantified using a GC-MS method.

### PBPK model for MPH and RA

**Figure 2:**
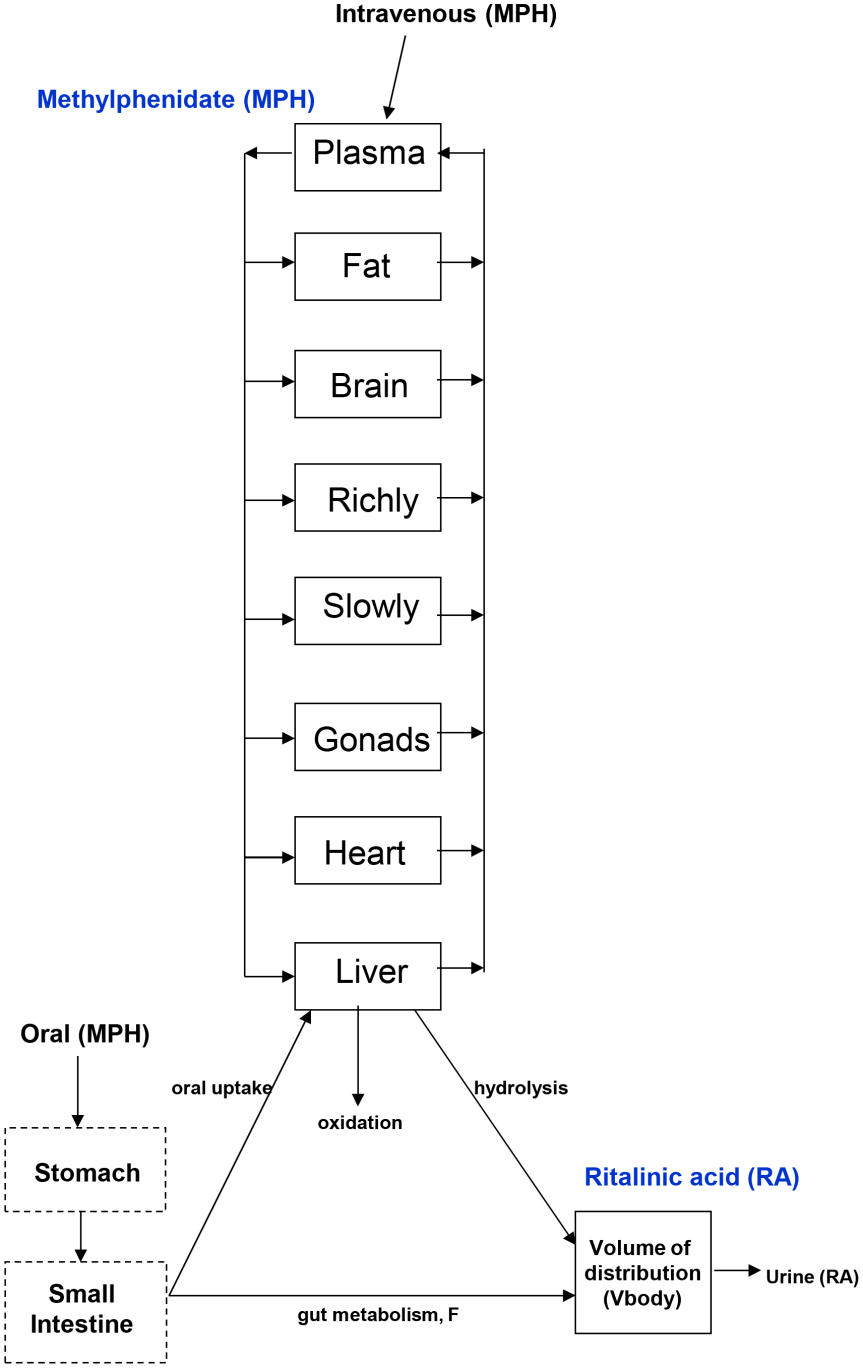
Schematic depicting the PBPK model for MPH and its primary metabolite RA. Two identical 8-compartment models were constructed for *d-* and *l-*MPH and two identical one-compartment models were built for *d-* and *l-*RA. MPH was given intravenously or orally. In humans, MPH is metabolized predominantly by hydrolysis to pharmacologically inactive RA, which is subsequently excreted into urine.

Two duplicate 8-compartment PBPK models for *d-* and *l-*MPH enantiomers (plasma, fat, brain, richly perfused, slowly perfused, gonads, heart, and liver) were constructed for children and adult humans as well as juvenile and adult rhesus monkeys. Competitive metabolic inhibition of each MPH enantiomer was described in the liver giving rise to formation of the primary metabolites, *d-* and *l-* ritalinic acid. Ritalinic acid was described using one compartment for each enantiomer (Figure 2). The selection of compartments for MPH was based on the metabolism and disposition as well as the potential target tissues of MPH (e.g. gonads, brain, and heart) [12], [26], [56]. The *d-* and *l-*RA enantiomers lack pharmacological activity [57] and were simply described without tissue compartments. The decay of MPH from systemic circulation and tissues occurred at a similar rate [58]–[60]. As such, distribution of MPH to the tissues was assumed to be flow limited in the current model. The simulations were performed using acsIXtreme, version 3.0.2.1 (The Aegis Technologies Group, Inc., Huntsville, AL).

#### Tissue to plasma partition coefficients

Tissue-to-plasma partition coefficients for MPH were estimated using a mechanistic model [61] based on tissue composition and compound specific parameters. A single pKa value of 9.51 (moderate base), a logP value of 2.31, and a logD value of 0.24 at pH 7.4 were predicted for MPH using the ACD Lab Solubility Suite (Advanced Chemistry Development, Inc., Toronto, Ontario, Canada). These properties were then used to estimate tissue and plasma partition coefficient values. Due to lack of available information, partition coefficients derived in one animal species have been applied for other animal models as well as humans, and vice versa [62], [63]. As such, in the current model, tissue-plasma partition coefficient values for liver, brain, and heart were determined based on monkey tissue composition, while those for fat and gonads were set to the values estimated in rats. Tissue-to-plasma partition coefficients for richly perfused and slowly perfused tissues were set to the values of the liver and the muscle estimated in monkeys (Table 1). The estimated tissue-to-plasma partition coefficients were generally consistent with those derived from the terminal phase of plasma and tissue pharmacokinetics studies in rats [58]–[60].

**Table 1:** Estimated tissue-plasma distribution coefficients for MPH.

Tissues	Partition coefficients (tissue/plasma)
Fat (Pfat)	1.79
Brain (Pbrain)	6.07
Richly perfused (Prich)	5.66
Slowly perfused (Pslow)	2.47
Gonads (Pgonads)	3.12
Heart (Pheart)	2.19
Liver (Pliver)	5.66

#### Physiological model parameters

The physiological model parameters PBPK for adult humans were derived from the literature (Table 2). For children, volume of tissues as a function of age, except for the fat, were estimated using the equations developed by Haddad et al. [64]. The volume for the fat was predicted using a TABLE function based on the calculated adipose tissue volumes of 3.68, 6.25, and 11.49 L for children 6, 10, and 14 yrs of age, respectively, for which lipid contents of all other tissues were excluded [65]. Changes in plasma volumes during growth were predicted as a function of body weight [66]. The reported cardiac output (QC, L/h) values in children, aged 0.5 to 15 years [67], [68], were assembled and fitted with regression equations to describe the relationship between QC (L/h) and age (year) in males and females:
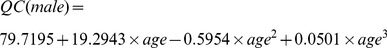
(1a)


**Table 2:** Physiological model parameters.

Parameters	Adult Humans	Children	Monkeys^e^	References
**Body weight, BW (kg)**	Study specific	Study specific	Study specific	Experimental data or [105] ^H^ [106] ^C^
**Cardiac output, QCC (L/h/kg^0.75^)**	15.87	Calculated using Eq.1	18.96	[63] ^H,M^
**Blood flows (fraction of cardiac output)**				
Fat (QFC)	0.053/0.091^a^	0.053/0.091^b^	0.02	[68] ^H^ [80] ^M^
Liver (QLC)	0.24	Calculated	0.194	[63] ^H^ [65] ^C^ [63] ^M^
Brain (QBC)	0.11	Calculated using Eq.2	0.07	[107] ^H^ [80] ^M^
Heart(QHC)	0.038/0.047^a^	0.038/0.047^b^	0.055	[67] ^H^ [80] ^M^
Gonads (QGC)	0.00054/0.00022^a^	0.00054/0.00022^b^	0.00054/0.00022^b^	[68] ^H^
Richly perfused (QRC)	0.76-QLC-QGC-QBC	0.76-QLC-QGC-QBC	0.76-QLC-QGC-QBC	
Slowly perfused (QSC)	0.24-QFC-QHC	0.24-QFC-QHC	0.24-QFC-QHC	
**Tissue volumes (fraction of body weight)**				
Plasma(VPC)	0.0435	Calculated	0.0627	[63] ^H^ [66] ^C^ [63] ^M^
Fat (VFC)	0.213/0.327^a^	Calculated	0.179/0.199^a^	[63] ^H^ [65] ^C^ [108] ^M^
Liver (VLC)	0.026	Calculated	0.03	[107] ^H^ [64] ^C^ [80] ^M^
Brain (VBC)	0.02	Calculated	0.018	[107] ^H^ [64] ^C^ [80] ^M^
Heart(VHC)	0.0045/0.0042^a^	Calculated	0.0037	[67] ^H^ [64] ^C^ [80] ^M^
Gonads (VGC)	0.0007/0.0027^a^	Calculated	0.0007/0.0027^b^	[63] ^H^ [64] ^C^
Richly perfused (VRC)	0.33-VLC-VPC-VGC-VBC	0.33-VLC-VPC-VGC-VBC	0.33-VLC-VPC-VGC-VBC	
Slowly perfused (VSC)	0.60-VFC-VHC	0.60-VFC-VHC	0.60-VFC-VHC	

a male/female; ^b^set to adult human values; ^e^for both adult and juvenile monkeys; ^H^adult humans;^ C^children; ^M^monkeys.



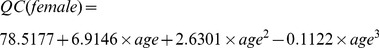
(1b)


Liver blood flow rates (Qliver, L/h) at different ages were predicted using a TABLE function based on the reported average liver blood flow rates of 325, 665, and 915 ml/min for children aged 4–8, 9–12, and 13–15 years, respectively [69]. Blood flow rates (L/h) to the brain (Qbrain) were estimated using the following best fit equation as a function of age (0.5 to 15 yrs) derived from the reported values [67]:
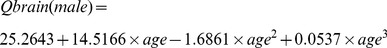
(2a)




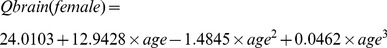
(2b)


Because of the lack of information on age-specific blood blows to the heart, fat, and gonads, the same percentages of cardiac output that were reported for adults were adopted for children.

Physiological PBPK model parameters for adult and juvenile rhesus monkeys were derived from the literature for adult monkeys, with the exception of gonads, which were taken from the human literature (Table 2).

### Model Development: Adult Humans

#### MPH: hepatic metabolism

In adult human livers, the majority (approximately 80%) of MPH is metabolized by hydrolysis resulting in the formation of RA [18], while the remaining is subject to oxidation [13], [18]. The stereoselective hydrolysis (Rmet_liver, µg/h) of *d*- and *l*-MPH was described using a Michaelis-Menten equation representing the competitive binding to the hCES1A1 enzyme between *d*- and *l*-MPH [16], [29]:
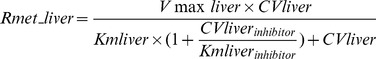
(3)


The Michaelis constants for *d*- and *l*-MPH (Kmliverd and Kmliverl, µg/L) were set equal to the reported Km values of 27,600 and 10,172 µg/L, experimentally determined using the recombinant human CES1A1 enzyme [16] (Table 3). CVliver is the venous plasma concentration leaving the liver for one isomer (CVliverd and CVliverl, µg/L) and CVliver_inhibitor_ is the venous plasma concentration leaving the liver for the inhibiting isomer (CVliverl and CVliverd, µg/L). Kmliver_inhibitor_ represents the dissociation constant for the inhibiting isomer, set to the Kmliver value of that isomer (Kmliverl and Kmliverd, µg/L). Vmaxliver (µg/h) is a scaled maximum hepatic reaction velocity, described as the product of the maximum hepatic reaction velocity constant (VmaxliverdC and VmaxliverlC, µg/h/kg^0.75^, for *d*- and *l*-MPH) and the body weight (BW)^0.75^. VmaxliverdC and VmaxliverlC (µg/h/kg^0.75^) were initially derived from the calculated *in vitro* maximal velocity of 38,496 and 78,111 ng/h/mg protein, which were obtained based on the reported *in vitro* catalytic constant values (Kcat, 0.165 min^−1^ and 0.335 min^−1^ for *d*- and *l*-MPH) using the recombinant human CES1A1 enzyme [16]. The *in-vitro in-vivo* extrapolations (IVIVE) were performed by accounting for microsomal protein content of the liver (39.19 mg microsomal protein/g liver [70]) and model predicted average liver weight (2.06 kg) for healthy men 18–30 years old [4], and estimated body weight of 74.8–84.02 kg [38]. A relative activity factor of 0.22, determined as the ratios of the imidapril hydrolase activity in human liver microsomes to the value for recombinant human CES1A1 enzyme [71], was considered to bridge the gap between the recombinant enzyme and native liver microsomes. Optimization of IVIVE derived VmaxliverdC and VmaxliverlC values was attempted using the NelderMead algorithm by simultaneous fitting to plasma concentration time courses of *d-* and *l-*MPH following iv dosing of 10 mg MPH in healthy adult men over a period of 16 h [4]. A convergence of values for VmaxliverdC and VmaxliverlC could not be achieved. Hence, the derived initial VmaxliverC values for *d-* and *l-*MPH were eventually adjusted manually (1.5 fold) to attain the best agreement between prediction and observed plasma *d-* and *l-*MPH concentrations (Table S1).

**Table 3:** Chemical specific model parameters.

Parameters	Adult Humans		Children	Adult Monkeys		Juvenile Monkeys		Method of calibration
	IV	Oral	Oral	IV	Oral	IV	Oral	
**Methylphenidate (MPH)**								
**Hepatic hydrolysis**								
Kmliverd (µg/L)	27,600	27,600	27,600	27,600	27,600	27,600	27,600	[16]
Kmliverl (µg/L)	10,172	10,172	10,172	10,172	10,172	10,172	10,172	[16]
VmaxliverdC (µg/h/kg^0.75^)	38,000	38,000	38,000^a^	38,000^a^	38,000^a^	350,000	350,000	[16] H and visual fit
VmaxliverlC (µg/h/kg^0.75^)	90,000	90,000	90,000^a^	90,000^a^	90,000^a^	700,000	700,000	[16] H and visual fit
**Hepatic oxidation**								
KmetdC and KmetlC (L/h/kg^0.75^)	0.7	0.7	0.7^a^	0.7^a^	0.7^a^	70	70	[18] H and visual fit
**Gastric emptying**								
GEdC and GElC (1/h/kg^−0.25^)	/	3.5	3.5^a^	/	2.34	/	2.34	[78] H [63] ^M^
**Oral uptake, from small intestine to liver**								
K3dC and K3lC (1/h/kg^−0.25^)	/	1.293	1.293^a^	/	1.293^a^	/	1.293^a^	Optimization^H^
**Gut metabolism**								
K5dC (1/h/kg^0.75^)	/	0.042	0.042^a^	/	1.05	/	/	Optimization^H^ and visual fit
K5lC (1/h/kg^0.75^)	/	1.426	0.1	/	35.65	/	/	Optimization^H^ and visual fit
F	/	0.8	0.8^a^	/	0.8^a^	/	/	[18] H
**Ritalinic acid (RA)**								
**Volume of distribution** (VbodyC, L/kg)	0.600	0.600	0.572	0.693	0.693	0.693	0.693	Set to total body water volume: [80] H ^,M^ [84], [85] ^C^
**Urinary excretion**								
Ku_RAdC (L/h/kg^0.75^)	0.305	0.305	0.305^a^	0.305^a^	0.305^a^	0.305^a^	0.305^a^	Optimization^H^
Ku_RAlC (L/h/kg^0.75^)	0.168	0.168	0.168^a^	0.168^a^	0.168^a^	0.168^a^	0.168^a^	Optimization^H^

H adult humans;^ C^children; ^M^monkeys; ^a^set to adult human values.

The oxidation metabolic pathways for the MPH enantiomers in the liver were described using clearance terms (KmetdC and KmetlC, L/h/kg^0.75^). This metabolic pathway for each enantiomer was constrained to yield an upper bound equal to 20% of the total dose metabolized in the liver [18]. The enzymes responsible for the hepatic oxidation of MPH have not yet been identified. CYP2D6 is known not to be involved [72].

#### MPH: Oral uptake and gastrointestinal (GI) tract metabolism

The use of the hepatic metabolic constants derived from iv dosing of adult humans consistently overestimated the plasma levels of MPH following oral administration, even with a small first order oral uptake constant. The metabolism of MPH in the GI tract by hydrolysis and oxidation was introduced into the human PBPK MPH model to achieve better predictions of observed plasma MPH concentrations following oral administration [24], [29]–[33]. The rationales for the inclusion of GI tract metabolism are as follows.

Though the predominant human CES1 enzyme identified for the hydrolysis of MPH was found primarily in human livers, expression of CES1 is also present in the human GI tract as identified by Northern blots [73], [74]. In addition, hydrolysis of flurbiprofen derivatives (flurbiprofen hydroxyethyl ester and hydroxypropyl ester), which are excellent substrates for hCES1 but not for hCES2, has been reported in human small intestine microsomes [75]. Other interesting observations suggest that the pharmacokinetics of the orally administered MPH is much less straightforward than iv administration. Higher plasma levels of *d*-MPH compared to *l*-MPH were observed immediately after oral administration (0.5 h), but not apparent until 1.5 h after iv administration [4]. Also, a distortion of the enantiomeric ratio for RA (*l*>>*d*) was observed in both human plasma and urine samples in the first 2 h after oral but not iv administration [28], [58]. Such route-dependent discrepancies found in the first 2 hours after dosing suggested the potential enantioselective presystemic metabolism of orally administered MPH in the GI tract. Expression of CYP enzymes has also been reported in human small intestines [76], although the enzymes responsible for the oxidation of MPH have not been identified [72], [77]. As such, metabolism of MPH in the GI tract by hydrolysis and oxidation was considered in the model, which was crucial to improving model performance.

Following oral administration of MPH, gastric emptying of *d-* and *l-*MPH into the small intestine was described using first order gastric emptying constants (GEdC and GElC, 1/h/kg^−0.25^) set to a value of 3.5 1/h/kg^−0.25^ [63], [78]. The majority (80%) of orally administered MPH was excreted in urine. RA accounted for 80% of total urinary metabolites, and feces accounted for 3.3% [18]. MPH emptied from the stomach lumen into the small intestine lumen was assumed to be immediately available within enterocytes, where MPH is either rapidly absorbed into the portal blood supply [13] or metabolized in the GI tract as discussed above. The oral uptake of *d-* and *l-*MPH was described as a first order process (K3dC and K3lC, 1/h/kg^−0.25^), with no evidence for the stereospecific absorption [16]. To account for the metabolic degradation of *d-* and *l-*MPH in the gut, first-order terms (K5dC and K5lC, 1/h/kg^0.75^) were employed, of which, a fraction (F = 0.80, 80%) was assumed to undergo hydrolysis to form RA, and be immediately absorbed into the systemic circulation. The remaining fraction (1-F) was assumed subject to oxidation. Optimized oral uptake constants (K3dC and K3lC, 1/h/kg^−0.25^) and metabolism constants (K5dC and K5lC, 1/h/kg^0.75^) for *d-* and *l-*MPH were obtained by simultaneous fitting to plasma concentrations of *d*- and *l*-MPH in adult humans orally dosed with MPH at 0.3 mg/kg [29], [30] and 40 mg [31], as well as plasma concentrations of MPH and RA in adult men orally dosed with MPH at 20 mg [32], [33] and 0.15 and 0.3 mg/kg [24] (Table S1). Optimization was carried out using the NelderMead algorithm.

#### RA: formation, distribution and systemic clearance

The rate of MPH hydrolysis in the liver and the GI tract was set equal to the rate of RA formation. Given that RA is highly soluble in the aqueous medium [79], the volume of distribution for RA was set to the value of total body water volume (0.6 L/kg) in adult humans [80]. Optimized systemic clearance terms for *d-* and *l-*RA (Ku_RAdC and Ku_RAlC, L/h/kg^0.75^) were obtained by simultaneous fitting to the urinary excretion of *d*- and *l*-RA over a period of 16 h after iv dosing of 10 mg MPH in healthy adult men [28] (Table S1). Optimization was performed using the NelderMead algorithm.

### Model Development: Children

Stereoselective metabolism of MPH (*l*>>*d*) has been documented in children [7], [46]. Also the expression and activity of hCES1 toward MPH in liver S9 fractions did not differ between children (aged 6–18 years old) and the pooled adult human samples [45]. Thus, the maximum velocity constants for hepatic metabolism (hydrolysis) of *l*- and *d*- MPH (VmaxliverdC and VmaxliverlC, µg/h/kg^0.75^) in children were set to the adult values. With no information to assume otherwise, hepatic oxidation of *l*- and *d*-MPH was also assumed to occur in children. Though the predominant enzymes responsible for the oxidation of MPH have not yet been identified, studies have demonstrated that the CPY3A subfamily is the most important subfamily among the total P450 enzymes responsible for the biotransformation of drugs in the human liver, with CYP3A4 as the most abundant isoform [81], [82]. As such, the age-dependent oxidation of MPH in the liver was assumed to be represented by the ontogeny of CYP3A4 enzymes. Hepatic RNA and protein contents of CYP3A4 as well as its activity, characterized by 6β hydroxylation of testosterone, reached adult values after 1 year of age [83]. For this reason, the clearance terms describing the metabolism of *l*- and *d*-MPH via oxidation (KmetdC and KmetlC, L/h/kg^0.75^) in the liver of children were assumed to be the same as adults. Since no data are available to describe the age-dependent oral uptake and metabolism of MPH in the gut, model parameters specific for oral dosing describing oral uptake (K3dC and K3lC, 1/h/kg^−0.25^) and gut metabolism (K5dC and K5lC, 1/h/kg^0.75^) for children were assumed to be the same as adult humans.

Scaling of adult MPH-specific model parameters performed well for the prediction of plasma *d-*MPH levels, but consistently underestimated plasma *l-*MPH levels in boys administered 10 mg MPH orally [7], [46], even with a large oral uptake rate constant for *l*-MPH, suggesting that systemic clearance of *l*-MPH is slower in children compared with adults after oral dosing. Optimization of oral uptake and hepatic and gut metabolic parameters for MPH enantiomers was conducted using the NelderMead algorithm by simultaneous fitting to the plasma concentration time courses of *d-* and *l-*MPH in these children [7], [46], but consistent convergence of parameter values could not be achieved. Thus, with other MPH-specific parameters fixed to adult values, the first order constant (K5lC, 1/h/kg^0.75^) describing gut metabolism for *l*-MPH was decreased by approximately 14 fold to achieve better agreement between model predictions and observed plasma concentration time courses of *l*-MPH in these children [7], [46] (Table S2).

The scaled clearance terms representing urinary excretion of *l*- and *d*-RA (Ku_RAdC and Ku_RAlC, L/h/kg^0.75^) were set to the adult values because of a lack of the time course RA concentrations in plasma or urine of children. The volume of distribution for RA was set to the body water volume of 0.572 L/kg in children [84], [85].

As a consequence of uncertainty in model parameter specificity, this MPH PBPK model is fit for purpose. That is, model parameters were fitted to provide agreement between observation and prediction; other factors may be important, but are unknown and not described in the model.

### Model Development: Adult Monkeys

Due to the lack of experimental data to determine model parameters in adult monkeys, the development of the monkey PBPK model relied primarily on cross species extrapolation using allometric scaling, as demonstrated in other PBPK models [62], [63]. The model parameters for the adult human intravenously dosed with MPH were used for the adult monkey intravenously dosed with 0.3 mg/kg MPH (NCTR data). The volume of distribution (VbodyC, L/kg) for RA was set to the total body water volume of the adult monkey (0.693 L/kg) [80]. Adult human values for parameters describing hepatic hydrolysis (VmaxliverdC and VmaxliverlC, µg/h/kg^0.75^) and oxidation (KmetdC and KmetlC, L/h/kg^0.75^) were used to describe plasma MPH concentration time course in adult monkeys. Model parameters (Ku_RAdC and Ku_RAlC, L/h/kg^0.75^) representing the systemic excretion of *l-* and *d-*RA in adult monkeys were assumed to be the same as adult humans.

Describing the kinetics of MPH after oral administration of MPH in the adult monkey was not possible using adult human model parameters describing oral uptake and adult monkey model parameters derived from intravenous dosing of the adult monkey with MPH. To improve predictions, the first order constants describing the gut metabolism of *d*- and *l*-MPH (K5dC and K5lC, 1/h/kg^0.75^) were increased proportionally by 25 fold to visually fit the plasma MPH concentrations from 1 to 8 h (clearance phase) following a single oral dose of 0.3 mg/kg MPH in adult monkeys (NCTR data). The rationale for this re-parameterization was based on reports of more abundant intestinal expression of the CES1 enzyme [75], [86], [87] in monkeys than humans and more rapid hydrolysis of the CES1 substrates flurbiprofen derivatives (2 to 55 fold) [75] in monkey small intestines than humans.

### Model Development: Young Monkeys

Because of the lack of information on MPH disposition in young monkeys, the calibrated adult monkey model was extrapolated to describe MPH kinetics in young monkeys, as other PBPK models did [63], [88]. In sharp contrast to adult monkeys, plasma MPH was cleared more rapidly in young monkeys following iv administration [24]. To account for the observed rapid clearance of MPH in young monkeys, both maximum hepatic reaction velocity (VmaxliverdC and VmaxliverdC, µg/h/kg^0.75^) describing the hydrolysis and the clearance term (KmetdC and KmetlC, L/h/kg^0.75^) describing the oxidation in the liver derived from the adult monkey model were simultaneously increased by 10- and 100-fold to fit the plasma concentrations of MPH and RA following a single iv dose of 3 mg/kg MPH in young monkeys [24]. Due to the lack of information on urinary excretion of RA in young monkeys, parameters describing urinary excretion (Ku_RAdC and Ku_RAlC, L/h/kg^0.75^) and volume of distribution (VbodyC, L/kg) for young monkeys were set to adult monkeys values.

With hepatic metabolic constants for MPH and parameters describing systemic distribution and clearance for RA determined by iv dosing, plasma concentration time courses of MPH and RA after repeated oral doses of 2.5 and 12.5 mg/kg of MPH in juvenile monkeys (NCTR study) were predicted using gastric emptying (GEdC and GElC, 1/h/kg^−0.25^) and oral uptake (K3dC and K3lC, 1/h/kg^−0.25^) parameters derived from the adult monkey model. The metabolism of MPH in the gut was not considered necessary in young monkeys with respect to maintaining reasonable prediction of time course kinetics of MPH and RA in plasma. Research is needed to fully understand the metabolic pathways of MPH in the liver and the GI tract for both adult and young monkeys.

### Assessment of Model Performance

Because of the concern for children only the juvenile monkey and child MPH models were evaluated for their ability to predict measured plasma pharmacokinetic data sets for MPH and *d-* and *l-*MPH following oral administration of immediate-release MPH. To access model performance, the Root Mean Squared Error (RMSE) was calculated for data sets in the juvenile monkey reported in this paper (NCTR data) and from Johns Hopkins University [27] and for pediatric data sets reported by [7], [46]–[51].

Model performance was calculated as following:
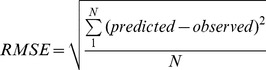
(4)where predicted is the model predicted plasma concentration and observed is the reported plasma concentration. N represents number of predictions and observations.

### Interspecies Extrapolation (Monkey to Human)

Juvenile male rhesus monkeys, 5 years of age, experienced a temporary decrease in circulating testosterone levels after chronic oral exposure to 2.5 mg/kg MPH and for 12.5 mg/kg MPH, a decrease in circulating testosterone levels along with impaired testicular descent, and reduced testicular volume [26]. Boys are more frequently diagnosed with ADHD than girls [89]. MPH is approved by the FDA for use in patients 6 years of age and older [13]. Given that the juvenile monkey toxicity data was in males, the developmental toxicity of MPH was extrapolated from male juvenile monkeys to boys between approximately 6 and 15 years of age. The model performance (RMSE) for the 12.5 mg/kg juvenile monkey dose group was judged inadequate for model predictions in humans (see Results).

PBPK derived oral human equivalent doses (HEDs) were only derived for the 2.5 mg/kg MPH juvenile monkey dose group. MPH HED values were calculated for the dosimetrics, maximum plasma concentration (Cmax, ng/mL) and daily area under the plasma MPH concentration curve (daily AUC, ng/mL*h per day). Preliminary simulations revealed no plasma accumulation of MPH in juvenile monkeys following a child’s therapeutic dosing schedule; while for children, a slight accumulation of plasma MPH levels was noticed with periodicity reached within 3 days. Thus, repeated daily oral dosing of MPH was simulated for 3–7 days to ensure steady state of MPH for both juvenile monkeys and children. Briefly, for juvenile monkeys, a one-week exposure for oral ingestion of MPH (2.5 mg/kg) occurred twice with a 4-h interval/day, 5 days a week, a dosing schedule utilized in the juvenile monkey toxicity study with MPH [26]. The dose metrics, Cmax and daily AUC calculated as the total AUC obtained from 1 week divided by 7 days (referred to as adjusted daily AUC, see Table S3), were recorded for MPH. Then simulations for children with repeated oral dosing of MPH twice a day, 7 days a week, with doses 4**h apart, were conducted with varying doses of MPH. The doses producing the equivalent internal dosimetrics (Cmax and daily AUC) of MPH at steady state (from day 4 to day 7) for children as those derived in the juvenile monkeys were determined as MPH HEDs.

In addition, model-predicted internal dose metrics (Cmax and daily AUC) in boys 6 and 15 years of age administered clinically recommended doses by the American Academy of Pediatrics (AAP) (0.3–0.8 mg/kg twice daily, taken 4 h apart) [90] for 1 week were compared with those obtained in juvenile rhesus monkeys experiencing delayed puberty as described above.

PBPK model code is contained in supplementary data (Text S2 and Text S3) and m files are available upon request.

### Sensitivity analysis

A time course local sensitivity analysis was implemented to assess the influence of parameter perturbations on model predictions of total MPH and *d*-MPH plasma concentrations over a 24-h period. A single oral dose of MPH (0.3 mg/kg) was simulated in both young and adult humans and monkeys. Normalized sensitivity coefficients (NSCs) were calculated using the partial derivatives of model output with respect to model parameters using the forward difference method, and normalized by both model output and model parameter [91]:
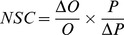
(5)where O is the model output (i.e. plasma concentration of total MPH or *d*-MPH), ΔO is the change in the model output, P is the value of the model parameter, and ΔP is the change in the parameter value. Model parameters were individually increased by 1% of their original values with all the other parameters held constant, except that simultaneous adjustment was conducted when evaluating the volumes and blood flow rates for slowly and richly perfused compartments to ensure mass balance. A positive NSC indicates a direct association between the model output and the corresponding parameter, while a negative NSC suggests the model output is inversely correlated with the specific parameter. Parameters with absolute NSC values greater than 0.1 were assumed to be sensitive.

## Results

### Model Calibration: Adult Humans

**Figure 3:**
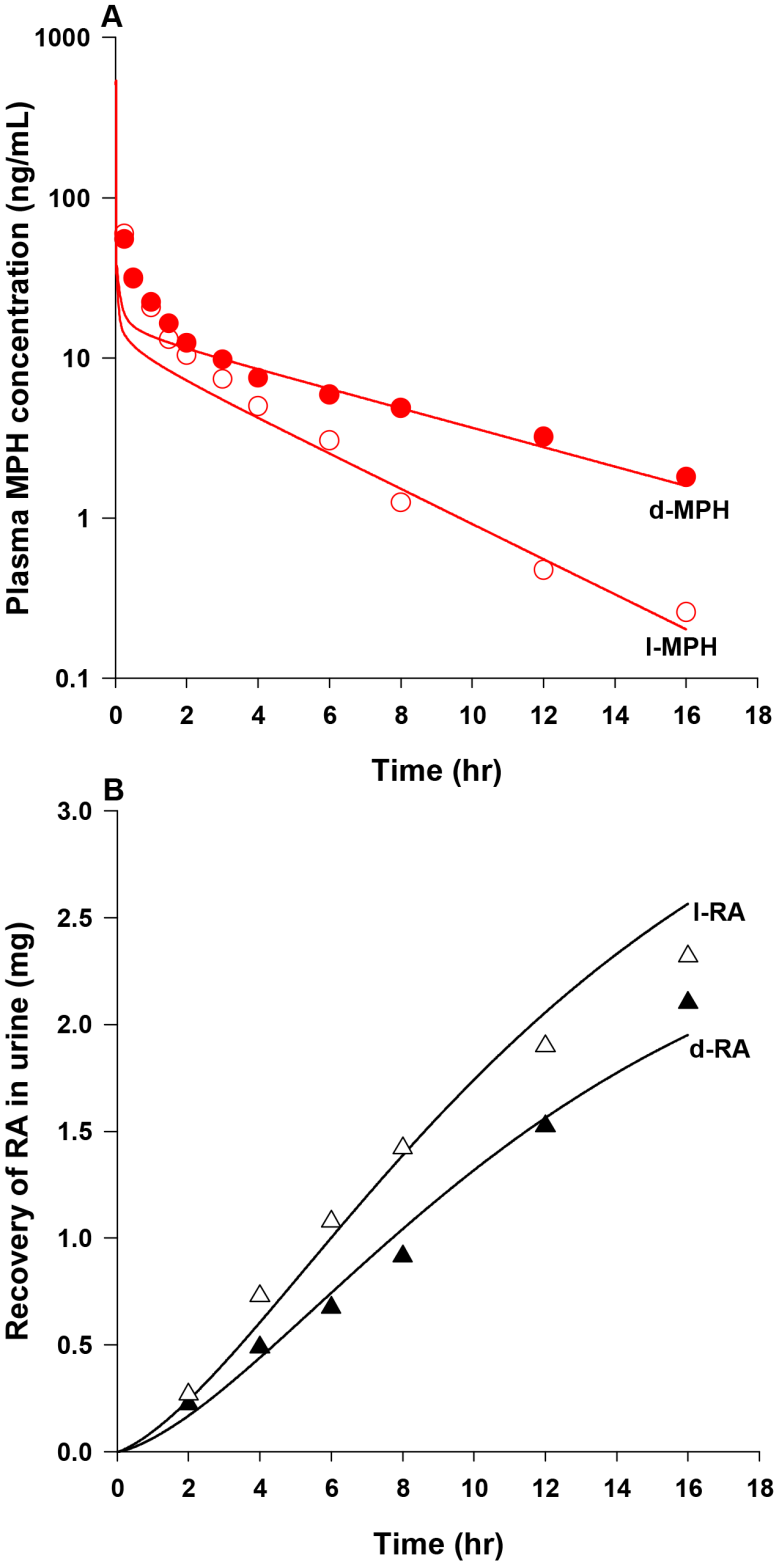
Plasma concentrations and urinary excretion data obtained after iv dosing of healthy adult men with MPH. Panel A: data represent model simulated (lines) and observed (circles) plasma concentrations of *d-*MPH (•) and *l*-MPH (○) after iv dosing with 10 mg MPH (n = 13) [4]; Panel B: data represent simulated (lines) and observed (triangles) urinary excretion of *d*-RA (▴) and *l*-RA (Δ) after iv dosing with 10 mg MPH (n = 9) [28]. Observed data were digitalized from graphs and are expressed as mean or mean ± SD based on the ability to digitalize: this applies to all figure legends unless otherwise specified.

For adult humans, enantioselective hydrolysis of *d*- and *l-*MPH in the liver was described using a Michaelis-Menten equation (Eq. 3). The Michaelis affinity constants, Kmliverd and Kmliverl, were set to values of 27, 600 and 10, 172 µg/L (Table 3), determined using the recombinant human CES1A1 enzyme [16]. The maximum hepatic reaction velocities, VmaxliverdC and VmaxliverlC, were slightly adjusted from their initial IVIVE derived values of 25,760 and 52, 270 µg/h/kg^0.75^ [16] to 38,000 and 90,000 µg/h/kg^0.75^ (Table 3) to get the best fit of plasma *d-* and *l-*MPH concentration profiles over a period of 16 h in healthy adult men (n = 13) following a single iv dose of 10 mg MPH [4] (Table S1). In conjunction with these parameters describing hepatic hydrolysis, hepatic oxidation of *d*- and *l-*MPH was described using clearance terms, KmetdC and KmetlC, of 0.7 L/h/kg^0.75^ to ensure approximately 20% of total hepatic metabolism occurs via oxidation [18]. With these hepatic metabolic parameters, plasma levels of *d-* and *l*-MPH in healthy adult men following a single iv dose of 10 mg MPH [4] were modestly under predicted in the first 2 hours after dosing, and for the remaining 14 hours model predictions agreed with observations (Figure 3A).

With model parameters describing hepatic hydrolysis (VmaxliverdC and VmaxliverlC) and oxidation (KmetdC and KmetlC) of *d-* and *l*-MPH resolved for adult humans, parameters representing systemic distribution (VbodyC) and clearance (Ku_RAdC and Ku_RAlC) of RA enantiomers were established. For *d-* and *l-*RA, because of their high water solubility [79], the volume of distribution (VbodyC) was set to a value of 0.6 L/kg equal to the total body water volume in adult humans [80]. Subsequently, the systemic clearance terms for *d*- and *l*-RA (Ku_RAdC and Ku_RAlC, 0.305 and 0.168 L/h/kg^0.75^, Table 3) were determined by an optimization algorithm using urinary excretion data of RA enantiomers collected over a period of 16 h in adult men after a single iv administration of 10 mg MPH (n = 9) [28] (Table S1). The calibrated model accurately reproduced the time course of urinary excretion profiles of *d*- and *l*-RA [28] (Figure 3B).

**Figure 4:**
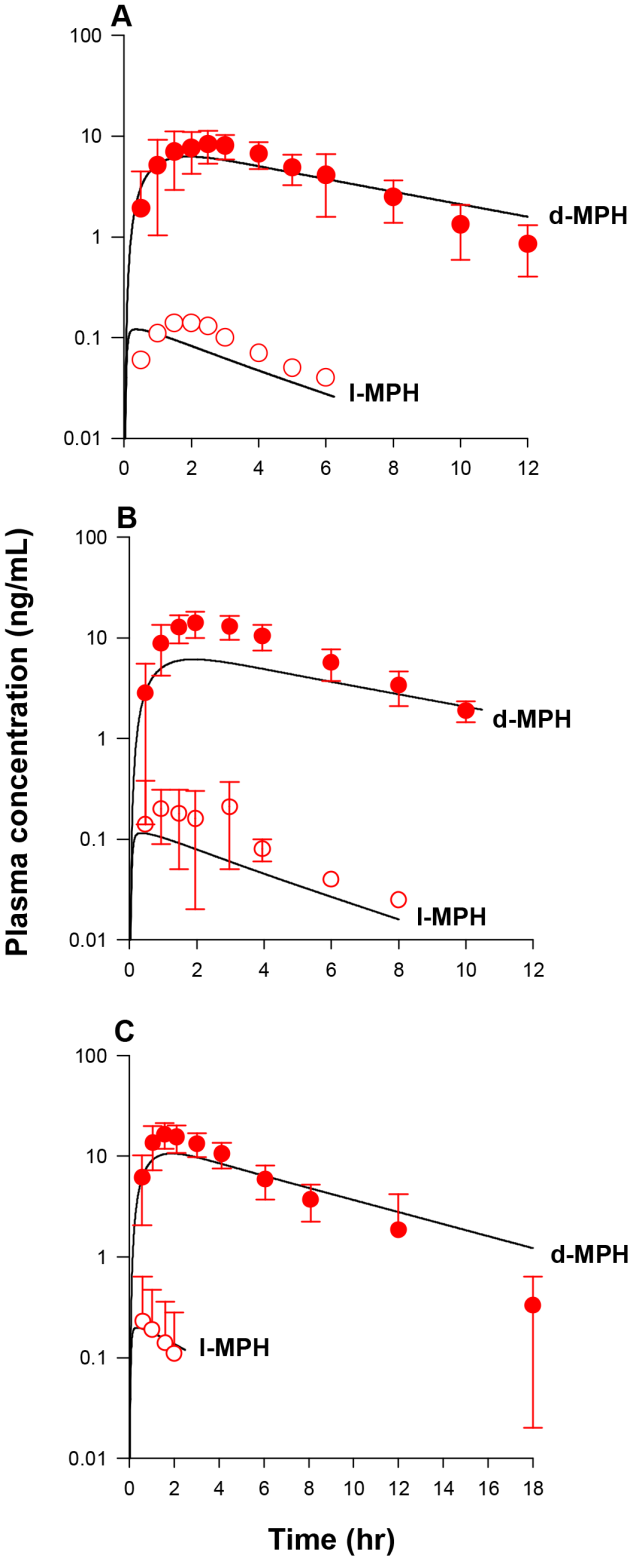
Plasma concentrations obtained after oral dosing of healthy adults with MPH. Panel A: data represent model simulated (lines) and observed (circles) plasma concentrations of *d-*MPH (•) and *l*-MPH (○) after oral dosing with 0.3 mg/kg MPH (n = 24) [29]; Panel B: Data as described for Panel A obtained after oral dosing with 0.3 mg/kg MPH (n = 19) [30]; Panel C: Data as described for Panel A obtained after oral dosing with 40 mg MPH (n = 21) [31].

**Figure 5:**
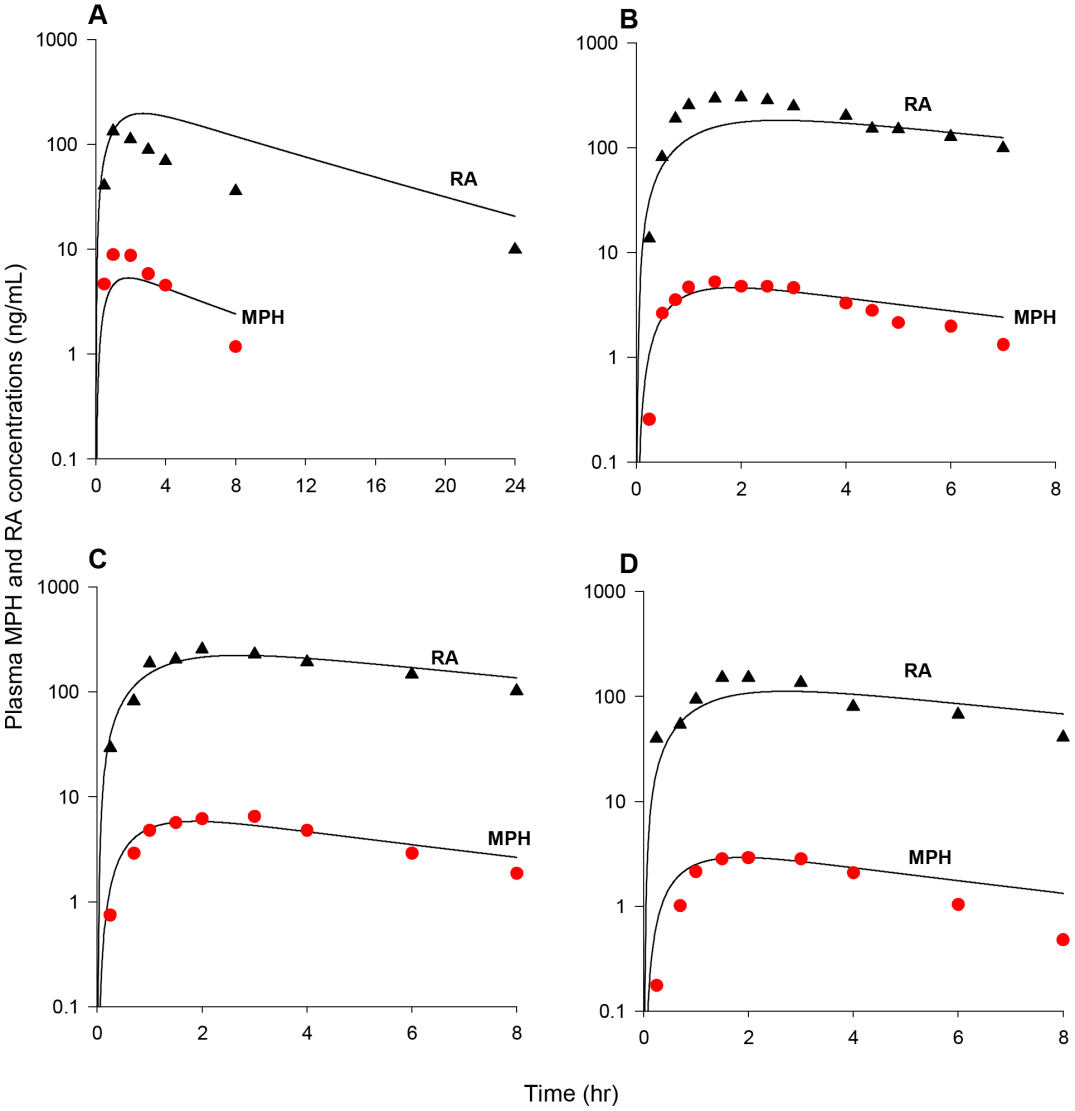
Plasma concentrations obtained after oral dosing of healthy adult men with MPH. Panel A: data represent model simulated (lines) and observed plasma concentrations of MPH (•) and RA (▴) after oral dosing with 20 mg MPH (n = 5) [32]; Panel B: Data as described for Panel A obtained after oral dosing with 20 mg MPH (n = 8) [33]; Panel C: Data as described for Panel A obtained after oral dosing with 0.3 mg/kg MPH (n = 10) [24]; Panel D: Data as described for Panel A obtained after oral dosing with 0.15 mg/kg MPH (n = 5) [24].

With enantiospecific model parameters describing hepatic hydrolysis (VmaxliverdC and VmaxliverdC) and oxidation (KmetdC and KmetlC) of *d-* and *l*-MPH as well as systemic distribution (VbodyC) and clearance (Ku_RAdC and Ku_RAlC) of *d-* and *l*-RA established for iv dosing in adult humans, model parameters specific for oral dosing of MPH representing oral uptake and gut metabolism were determined (Table 3). The gastric emptying first order constants for *d-* and *l*-MPH (GEdC and GElC) were set to the same value of 3.5 1/h/kg^−0.25^ [63], [78]. As discussed in the Methods, MPH emptied from the stomach lumen into the small intestine lumen was assumed to be immediately taken up by enterocytes. Within enterocytes, MPH is either rapidly absorbed into the portal blood supply [13] or metabolized in the GI tract, of which a fraction (F, 80%) was assumed to undergo hydrolysis to form RA and immediately be absorbed into the system. Optimization of oral uptake (K3dC and K3lC) and gut metabolism (K5dC and K5lC) constants was undertaken by seeking agreement with plasma concentration time courses of *d*- and *l*-MPH as well as MPH and RA in healthy adult humans orally dosed with MPH [24], [29]–[33] (Table S1), of which, time courses of plasma *d*- and *l*-MPH kinetics were collected in adult humans following a single oral dose of MPH at 0.3 mg/kg (n = 24) (Figure 4A) [29], 0.3 mg/kg (n = 19) (Figure 4B) [30], and 40 mg (n = 21) (Figure 4C) [31] over a period of time up to 18 h; and time courses of plasma MPH and RA kinetics were collected in adult men following a single oral dose of MPH at 20 mg (n = 5) (Figure 5A) [32], 20 mg (n = 8) (Figure 5B) [33], and 0.3 (n = 10) (Figure 5C) and 0.15 mg/kg (n = 5) (Figure 5D) [24] over a period of time up to 24 h. Optimized oral uptake constants (K3dC and K3lC) with values of 1.293 and 1.2931/h/kg^−0.25^ and gut metabolism terms (K5dC and K5lC) with values of 0.042 and 1.426 1/h/kg^−0.25^ (Table 3) along with other MPH-specific model parameters in general provided a good prediction of these measured *d-*MPH (Figures 4A–C) [29]–[31] and MPH (Figures 5A–D) [24], [32], [33] plasma kinetics with some exceptions: as shown in Figure 4C, the model overestimated plasma *d*-MPH level at 18 h by approximately 4 fold [31]; for plasma MPH levels, observations at 8 h for the studies of [32] (Figure 5A) and [24] (Figure 5D) were overestimated by 2–3 fold. Together with systemic distribution (VbodyC) and clearance (Ku_RAdC and Ku_RAlC) terms for *d-* and *l*-RA determined from iv dosing in adult humans, model predicted and measured plasma RA concentrations [24], [32], [33] (Figures 5A–D) were in general good agreement with the exception of one study [32], for which the model captured the time course of plasma RA concentrations for the first 2 hours post-dose, but slightly overpredicted observations for the remaining time points within 2–4 fold (Figure 5A).

### Model Calibration: Children

As described in the Methods, hepatic metabolic parameters representing hydrolysis (VmaxliverdC and VmaxliverlC, 38,000 and 90,000 µg/h/kg0.75) and oxidation (KmetdC and KmetlC, 0.7 and 0.7 L/h/kg^0.75^) of *d-* and *l-*MPH for children were set to adult values given that children display similar expression and activity of CES1 [45] and CYP3A4 [83] enzymes in the liver compared with adults. With no information to assume otherwise, MPH-specific model parameters representing oral uptake and gut metabolism (Table 3) were set to adult human values, except that the gut metabolism constant for *l*-MPH (K5lC) was visually adjusted from adult value of 1.426 to 0.1 1/h/kg^0.75^ to achieve a better fit to plasma concentration time course data of *l*-MPH over a period of up to 8 h in children following oral dose of 10 mg MPH [7], [46].

**Figure 6:**
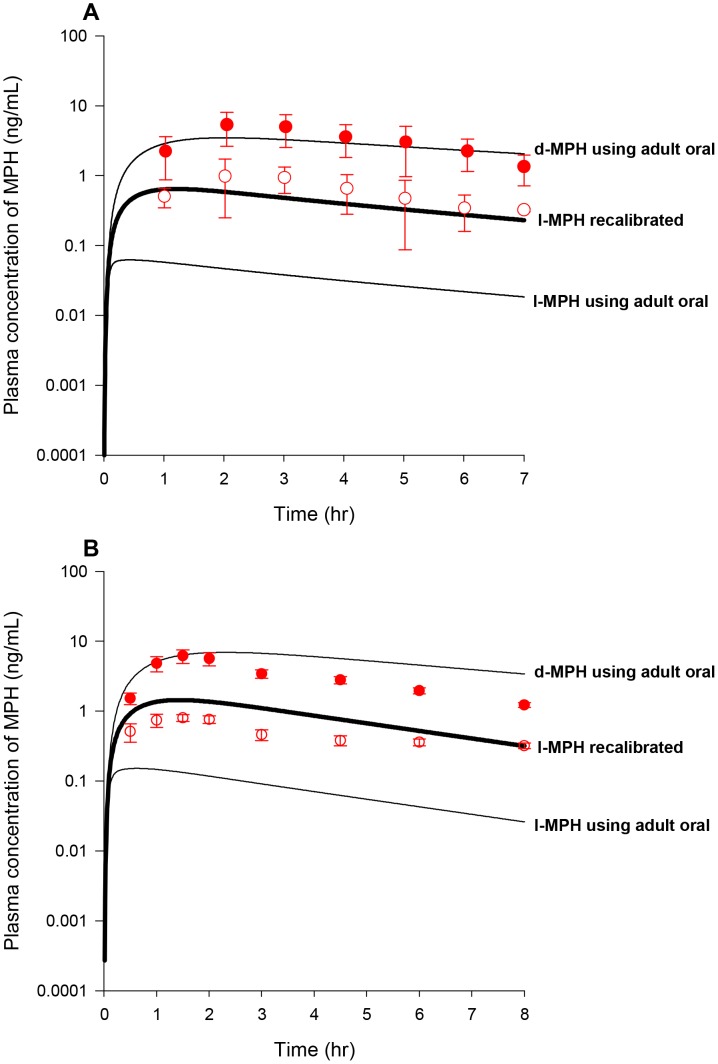
Plasma concentrations obtained after oral dosing of boys with MPH. Panel A: Data represent model simulated (lines) and observed (circles) plasma concentrations of *d*-MPH (•) and *l*-MPH (○) after oral dosing with10 mg MPH in boys with ADHD (n = 9) [7]; Panel B: Data as described for Panel A obtained after oral dosing with 10 mg MPH in boys with ADD (n = 5) [46]. Thin lines depict model simulations with MPH-specific model parameters set to adult values, and thick lines represent model predictions of plasma *l*-MPH concentrations with the calibrated children oral model, for which the value of K5lC describing gut metabolism of *l*-MPH was decreased from the adult value of 1.426 to 0.1 1/h/kg^0.75^, while other MPH-specific model parameters were set to adult values.

With the constant of K5lC recalibrated and other MPH-specific model parameters set to adult human values (Table 3), model predictions of plasma *d-* and *l-*MPH concentrations in boys with ADHD (n = 9, Figure 6A) and ADD (n = 5, Figure 6B) orally dosed with 10 mg MPH in general tracked experimental data except that in the study of [46] systemic clearance of *d*-MPH after 2 h was slightly faster than the model forecasted within a factor of 2–3 (Figure 6B). With all RA-specific model parameter values previously calibrated from the adult human model (Table 3), model estimated serum concentrations of RA for boys administered 10–15 mg MPH orally were in general consistent with the reported values (Table 4) [19].

**Table 4:** Observed and simulated serum RA concentrations in boys after oral administration of MPH.

Patient No.	Dose (mg/kg)	Time (h)	RA concentration (µg/L)	
			Observed	Simulated
1	0.64	2.0	275	413
2	0.47	1.5	315	261
3	0.37	1.1	250	150
4	0.29	2.25	285	203
5	0.25	2.5	165	178

### Model Calibration: Adult Monkeys

**Figure 7:**
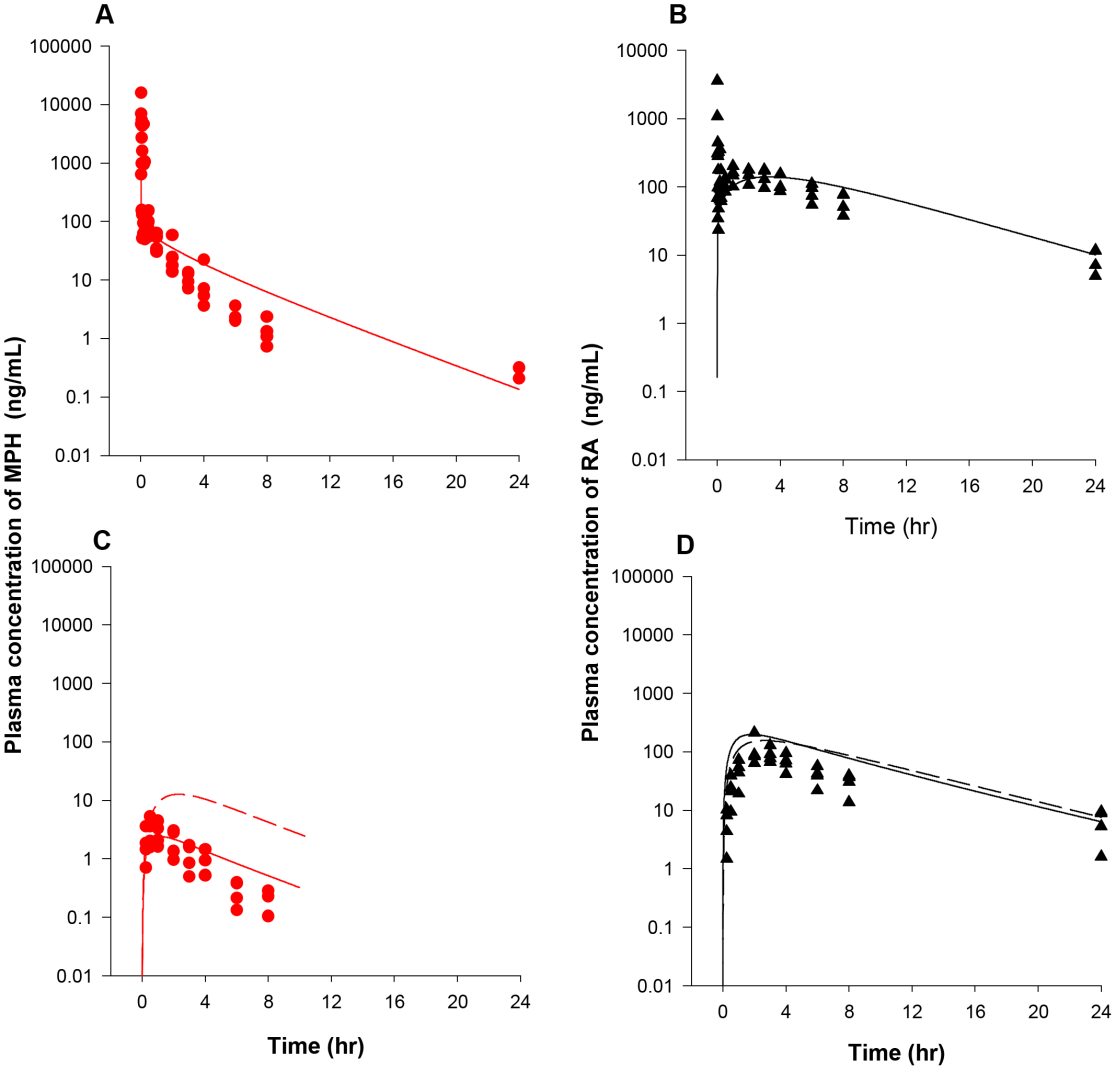
Plasma concentrations obtained after iv and oral dosing of adult monkeys with MPH (NCTR data). Panel A: Data represent model simulated (line) and observed (circles) individual plasma concentrations of MPH (•) after iv dosing with 0.3 mg/kg MPH (n = 4). One plasma sample at 6 h and two other plasma samples at 24 h contained non-quantifiable levels of MPH (0.1 µg/L limit of quantification, LOQ) [52]; Panel B: Data represent model simulated (line) and observed (triangles) individual plasma concentrations of RA (▴) after iv dosing with 0.3 mg/kg MPH (n = 4); Panel C: Data as described for Panel A obtained after oral dosing with 0.3 mg/kg MPH (n = 4). One plasma sample at 4 h and one plasma samples at 8 h contained non-quantitable levels of MPH [52]; Panel D: Data as described for Panel B obtained after oral dosing with 0.3 mg/kg MPH (n = 4). Dashed lines represent model predictions using kinetic model parameters derived from the adult human oral model, whereas solid lines depict model predictions using the calibrated adult monkey oral model, for which gut metabolism constants (K5dC and K5lC) were increased from adult human values of 0.042 and 1.426 1/h/kg^0.75^ to 1.05 and 35.65 1/h/kg^0.75^ for *d*-MPH and *l*-MPH.

Model parameters describing hepatic hydrolysis (VmaxliverdC and VmaxliverlC, 38,000 and 90,000 µg/h/kg^0.75^) and hepatic oxidation (KmetdC and KmetlC, 0.7 and 0.7 L/h/kg^0.75^) of *d-* and *l-*MPH for adult monkeys were set to adult human values (Table 3) given that monkeys exhibit similar hepatic CES1 [75] and P450 [92] activities as humans. Model simulations in general tracked the behavior of MPH in plasma over a period of 24 h for adult monkeys (n = 4) following iv administration of 0.3 mg/kg MPH (NCTR data), except that the measured levels were slightly overestimated at 6 h and 8 h, within a factor of 3 (Figure 7A). One plasma sample at 6 h and two other plasma samples at 24 h contained non-quantifiable levels of MPH (0.1 ng/mL limit of quantification, LOQ) [52]. Due to lack of information, values of Ku_RAdC and Ku_RAlC (0.305 and 0.168 L/h/kg^0.75^) representing systemic clearance of *d-* and *l-*RA were assumed to be the same as those of adult humans (Table 3). With volume of distribution for RA enantiomers (VbodyC, L/kg) set to a value of 0.693 L/kg equal to the total body water volume of the adult monkey [80], model simulations of plasma RA concentrations in adult monkeys (n = 4) following iv dosing of 0.3 mg/kg MPH (NCTR data) were in line with collected kinetic data, except that observations were under estimated at 0.02 h post dosing (Figure 7B).

With hepatic metabolic constants established for intravenously dosed adult monkeys, the gastric emptying first order constants for *d-* and *l-*MPH (GEdC and GElC) were set to the value of 2.34 1/h/kg^−0.25^ [63] (Table 3). Use of oral uptake constants (K3dC and K3lC, with a value of 1.293 1/h/kg^0.25^) and gut metabolism parameters (K5dC and K5lC, with values of 0.042 and1.426 1/h/kg^0.75^) derived from adult humans, the model captured MPH plasma concentrations following oral dosing of 0.3 mg/kg MPH in adult monkeys for the first two time points (0.25 and 0.5 h post-dose), but overestimated for the remaining time points (Figure 7C). To account for the reported greater intestinal expression and activity of CES1 [75], [86], [87] and P450 enzymes [92] in cynomolgus monkeys compared with humans, K5dC and K5lC values, representing small intestinal metabolism, were visually increased from adult human values of 0.042 and 1.426 1/h/kg^0.75^ to 1.05 and 35.64 1/h/kg^0.75^ to seek agreement with plasma levels of MPH from 1 to 8 h following oral dosing of 0.3 mg/kg MPH in adult monkeys (NCTR data). Using these calibrated model parameters, model predictions in general captured the kinetic behavior of MPH for the first 4 h after dosing, with one plasma sample contained non-quantifiable level of MPH at 4 h (Figure 7C). Model simulated plasma levels of MPH at 6 and 8 h post dosing were slightly higher than the measured levels, with 1 of 4 plasma samples at 8 h containing non-quantifiable MPH level. Using systemic distribution (VbodyC) and clearance parameters (Ku_RAdC and Ku_RAlC) for RA enantiomers determined from adult monkey iv dosing, simulations of plasma RA concentrations somewhat overestimated experimental data in adult monkeys after oral dosing of 0.3 mg/kg MPH (NCTR data) but maintained the general profile of the time course for plasma RA levels (Figure 7D).

### Model Calibration: Young Monkeys

**Figure 8:**
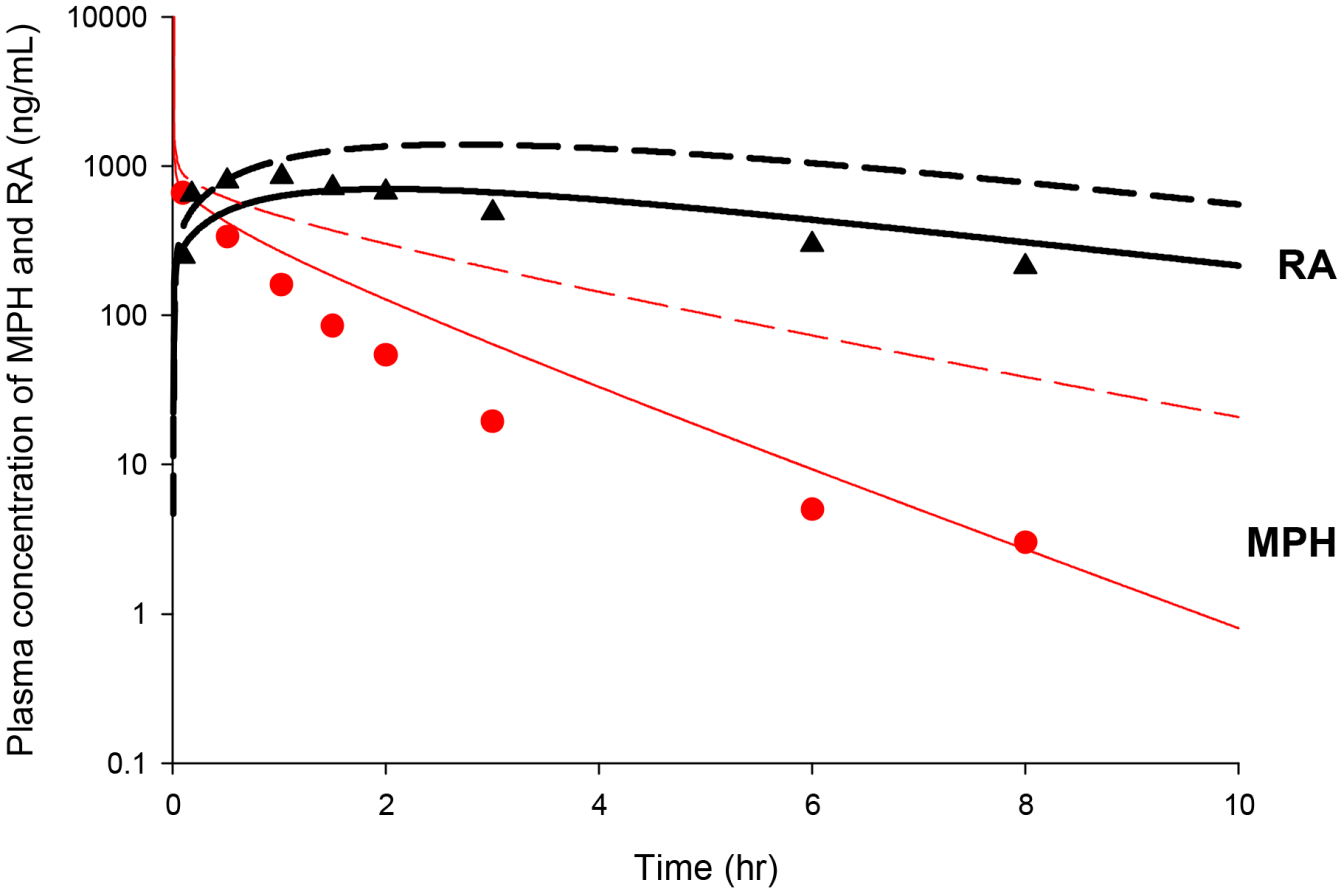
Plasma concentrations obtained after iv dosing of juvenile monkeys with MPH. Data represent model simulated (thin lines for MPH and thick lines for RA) and observed plasma concentrations of MPH (•) and RA (▴) after iv dosing with 3 mg/kg MPH (n = 5) [24]. Dashed lines represent model simulations using hepatic metabolic constants derived from the adult monkey iv model, whereas solid lines depict model predictions using the calibrated juvenile monkey iv model, for which maximum metabolic constants (VmaxliverdC and VmaxliverlC) describing hepatic hydrolysis and clearance terms describing hepatic oxidation (KmetdC and KmetlC) for *d*-MPH and *l*-MPH were increased from adult values of 38,000 µg/h/kg^0.75^, 90,000 µg/h/kg^0.75^, 0.7 L/h/kg^0.75^, and 0.7 L/h/kg^0.75^ to 350,000 µg/h/kg^0.75^, 700,000 µg/h/kg^0.75^, 70 L/h/kg^0.75^, and 70 L/h/kg^0.75^, respectively.

Scaling of adult monkey constants describing hepatic hydrolysis (VmaxliverdC and VmaxliverlC) and oxidation (KmetdC and KmetlC) largely overestimated plasma MPH and RA concentrations in juvenile monkeys after a single intravenous dose of 3 mg/kg MPH [24] (Figure 8). Simultaneous adjustment of these parameters was undertaken to seek a better fit to plasma kinetics for both MPH and RA in these juvenile monkeys [24], with parameters describing systemic distribution (VbodyC) and clearance (Ku_RAdC and Ku_RAlC) of RA enantiomers held to adult monkey values (Table 3). With VmaxliverdC, VmaxliverlC, KmetdC, and KmetlC values visually fitted to 350,000 µg/h/kg^0.75^, 700,000 µg/h/kg^0.75^, 70 L/h/kg^0.75^ and 70 L/h/kg^0.75^, respectively (Table 3), model predicted plasma MPH and RA concentrations were in general agreement with reported data [24], except for mild overestimations of MPH levels at 1.5, 2 and 3 h within a factor of 2–3 (Figure 8).

**Figure 9:**
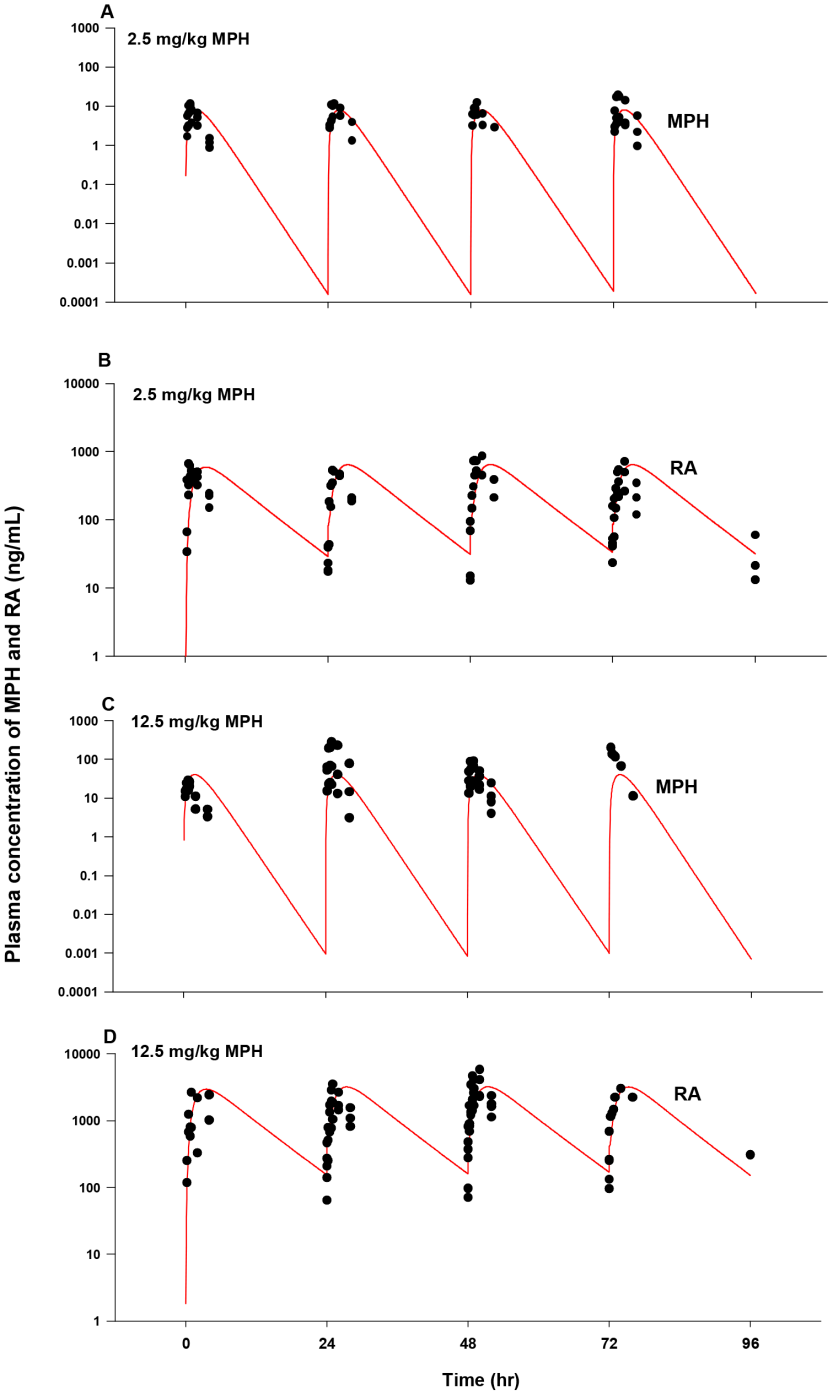
Plasma concentrations obtained after repeated oral dosing of juvenile male monkeys with MPH (NCTR data). Panel A: Data denote representative model simulated (lines) and observed (circles) individual plasma concentrations of MPH (•) after repeated oral dosing with 2.5 mg/kg MPH (n = 1–4 at each time point); Panel B: Data depict representative model simulated (lines) and observed (circles) individual plasma concentrations of RA (•) after repeated oral dosing with 2.5 mg/kg MPH (n = 1–4 at each time point). Measurements of plasma RA concentrations at pre-dose (approximately within 30 min of dosing) were combined with those at 24 h from previous dose; Panel C: Data as described for Panel A obtained after oral dosing with 12.5 mg/kg MPH (n = 1–4 at each time point); Panel D: Data as described for Panel B obtained after oral dosing with 12.5 mg/kg MPH (n = 1–4 at each time point). MPH was administered twice a day, 4 h apart, five days a week (Monday to Friday) and kinetic studies were performed from Monday to Thursday. Kinetic profiles of MPH and RA for each individual monkey were followed on the same day of the week when quarterly blood sampling occurred over a 1 year period. On the day of blood collection, MPH was administered only once in the morning.

Repeated oral dosing simulations with MPH (2.5 and 12.5 mg/kg, twice with a 4 h interval/day, five days a week) in young monkeys (n = 1–4 for each time point, NCTR data) were conducted using model parameter values for hepatic metabolism of MPH (VmaxliverdC, VmaxliverlC, KmetdC and KmetlC) and systemic distribution (VbodyC) and clearance (Ku_RAdC and Ku_RAlC) of RA derived from intravenous dosing of young monkeys [24], and for MPH gastric emptying (GEdC and GElC) and oral uptake (K3dC and K3lC) terms, set to adult monkey values (Table 3). Metabolism of MPH in the gut was not considered. Model predictions of plasma MPH and RA kinetics were in general agreement with observations for both low dose (2.5 mg/kg) and high dose (12.5 mg/kg) groups (Figures 9A–D) with a few exceptions: the model somewhat underestimated MPH levels within 1 h post dosing for the 12.5 mg/kg dose group except for Monday (Figure 9C) and overpredicted plasma RA levels at 4 h after dosing for both 2.5 and 12.5 mg/kg dose groups (Figures 9B and 9D). No apparent difference was noted for plasma MPH and RA kinetics collected periodically over the course of two years (Figure S1, Figure S2, Figure S3, and Figure S4).

### Model Evaluation: Adult Humans

**Figure 10:**
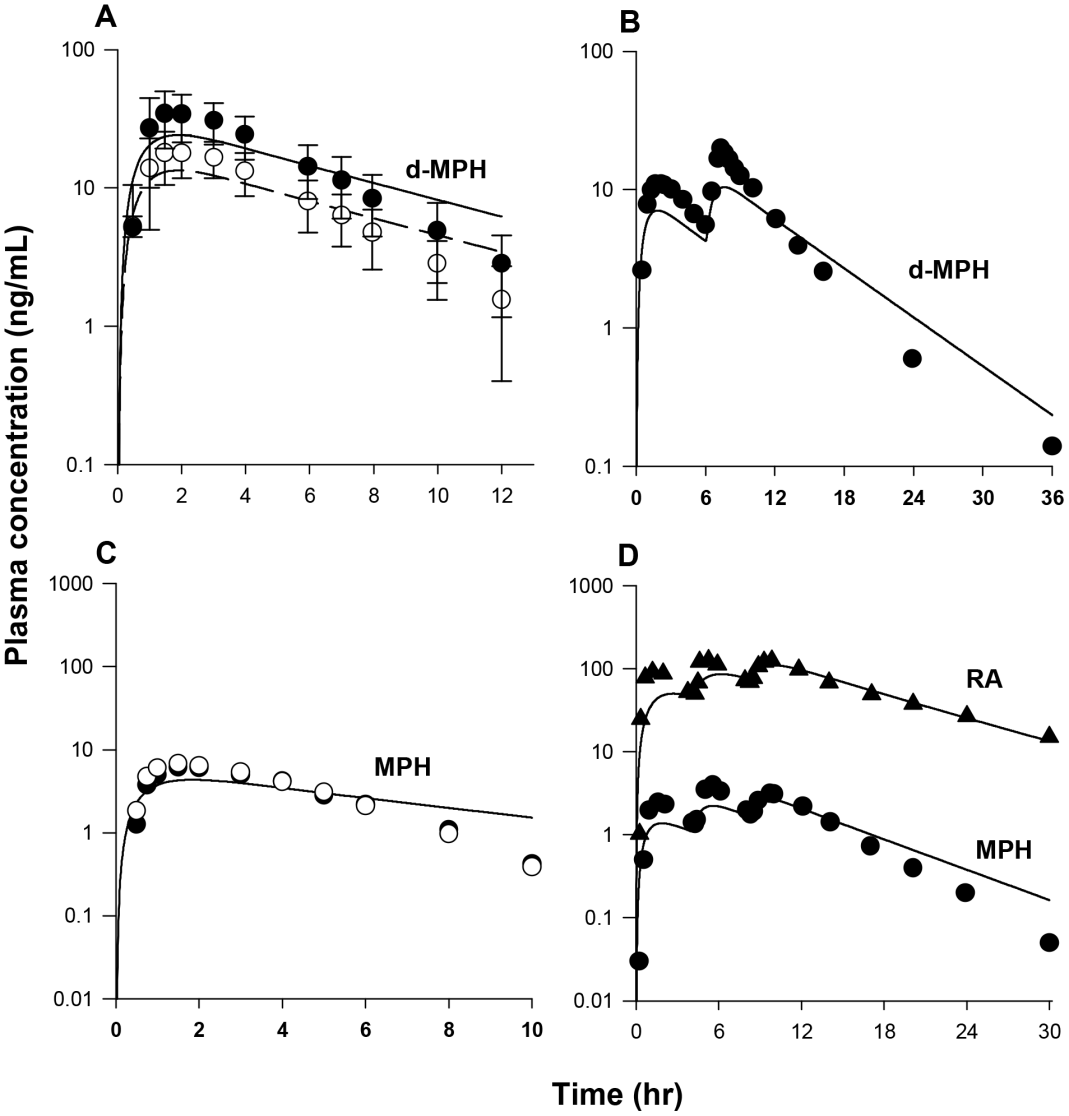
Plasma concentrations obtained after oral dosing of healthy adult humans with MPH. Panel A: Data represent model simulated (solid line, 90 mg MPH and dashed line, 50 mg MPH) and observed (circles) plasma concentrations of *d*-MPH after oral dosing with 90 mg (•) and 50 mg(○) MPH (n = 49) [34]; Panel B: Data represent model simulated (line) and observed (circles) plasma concentrations of d-MPH (•) after two repeated oral dosing with 30 mg/kg MPH, taken 6 h apart (n = 28) [35]; Panel C: Data represent model simulated (line) and observed (circles) plasma concentrations (○, test formulation; •, reference formulation) of MPH after oral dosing with 20 mg MPH (n = 20) [36]; Panel D: Data represent model simulated (line) and observed plasma concentrations of MPH (•) and RA (▴) after three repeated oral dosing with 5 mg/kg MPH, taken 4 h apart (n = 35) [37].

The calibrated adult human oral model was first evaluated against plasma *d*-MPH concentration time course data collected in healthy adults administered a single oral dose of 50 and 90 mg MPH [34], a dose level slightly higher than the doses (20–40 mg) used for model calibration (Table S1). The calibrated model provided a good prediction of plasma *d*-MPH kinetic behaviors, except that plasma *d*-MPH concentrations were slightly overestimated at 12 h within a factor of 2–3 for both dose groups (Figure 10A). Plasma *d-*MPH concentration time course data were also obtained from healthy adults given two repeated oral doses of 30 mg MPH, 6 h apart [35] (Table S1). The kinetic behavior of *d*-MPH over a period of 36 h was very well captured by the model (Figure 10B).

Figures 10C and 10D show model predictions and observations of plasma MPH concentrations in health adults administered a single oral dose of 20 mg MPH [36] and three repeated oral doses of 5 mg MPH taken 4 h apart [37], for which the time course of plasma RA concentration was also reported (Table S1). Model predictions of MPH plasma concentrations were adequate for both studies, except for a slight overestimation within a factor of 3–4 noticed at last time points for both studies (at 10 h for [36] and at 30 h for [37]). Model predictions of RA plasma concentrations were in excellent agreement with observed data [37].

**Figure 11:**
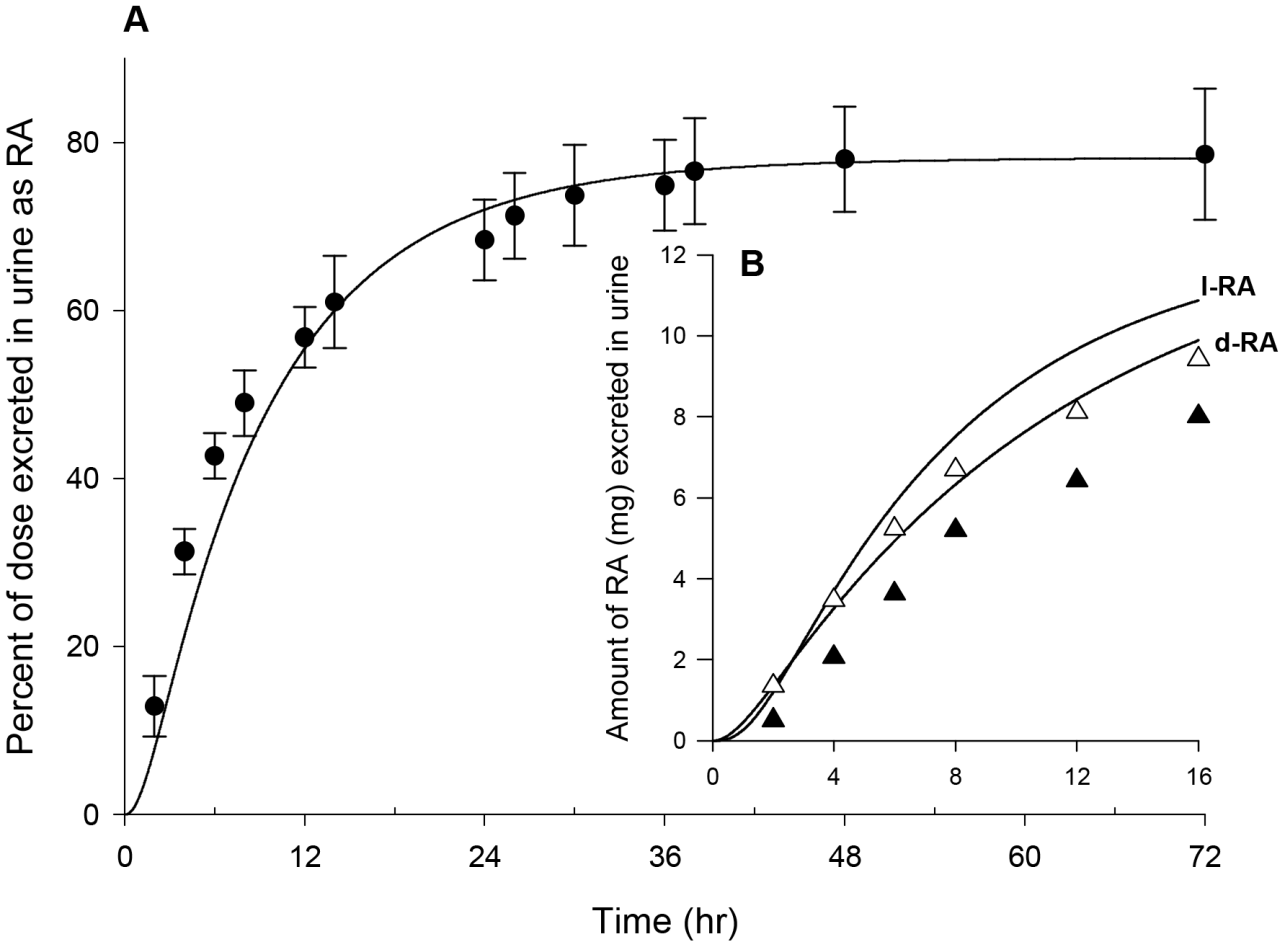
Urinary excretion data obtained after oral dosing of healthy adult men with MPH. Panel A: Data represent model simulated (line) and observed (circles) percentage of total dose excreted in urine as RA (•) after oral dosing with 20 mg MPH (n = 3) [20]; Panel B: Data represent model simulated (line) and observed (triangles) urinary excretion time courses of *d*-RA (▴) and *l*-RA (Δ) after oral dosing with 40 mg MPH (n = 9) [28].

The calibrated model was also tested against published urinary excretion data with oral MPH [20], [28] (Table S1). As shown in Figure 11A, the model accurately replicated the time course of urinary RA excretion over a period of 72 h after oral administration of 20 mg MPH in healthy men [20]. Urinary excretion time course for *d*- and *l*-RA over a period of 16 h following oral administration of 40 mg MPH in healthy men [28] was slightly overestimated within a factor of 1.5 (Figure 11B).

In addition, the calibrated adult human oral model was further evaluated against other published kinetic studies with MPH in healthy adults [38]–[43] (Table S1). With a few exceptions, the calibrated adult human model generally predicted plasma concentration time courses of MPH and RA collected in these single- and multiple-dose studies (Figure S5 and Figure S6).

### Model Evaluation: Children

**Figure 12:**
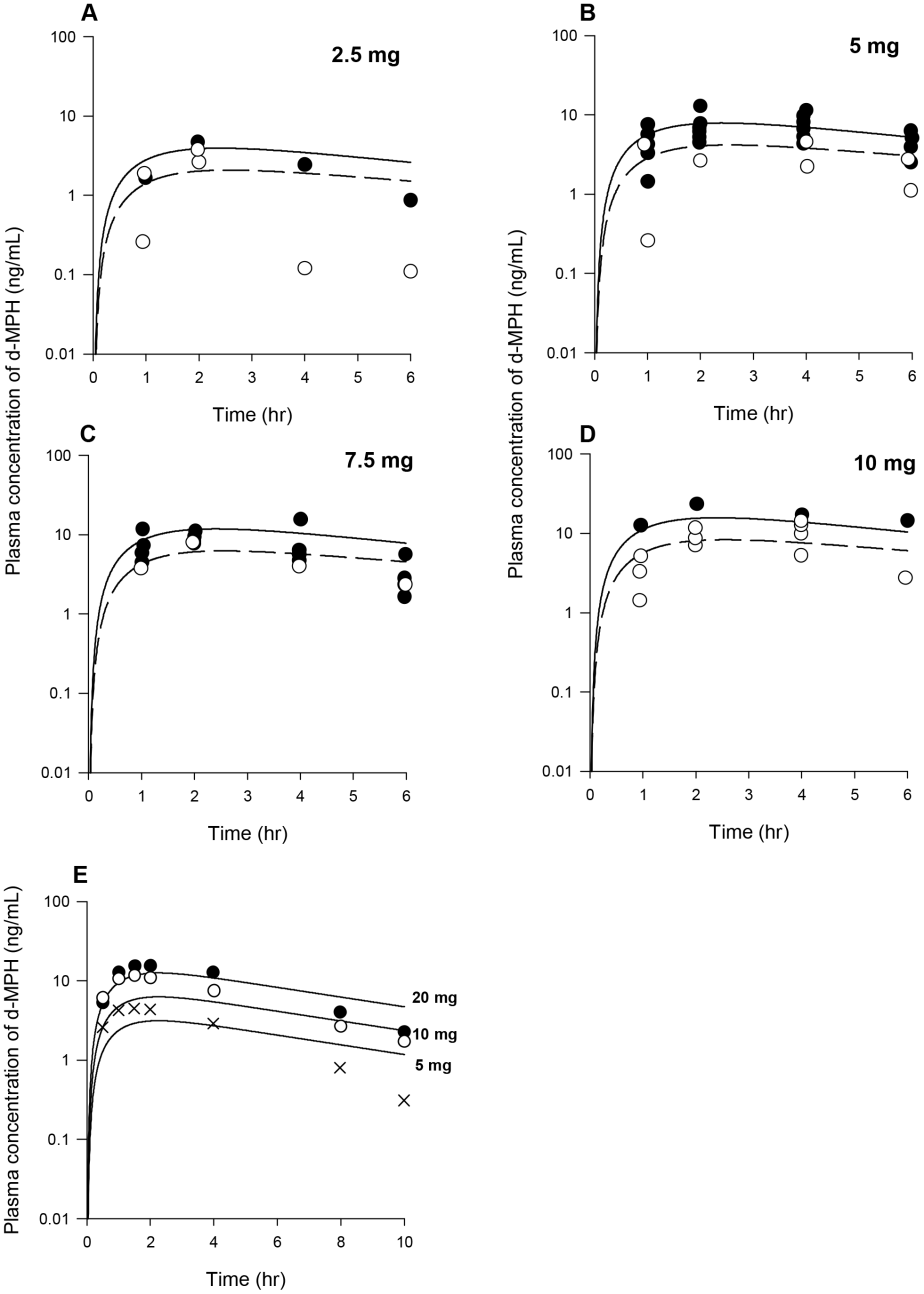
Plasma concentrations obtained after oral dosing of children with MPH. Panel A: Data represent model simulated (lines) and observed (circles) plasma concentrations of *d*-MPH after oral dosing with 2.5 mg MPH in preschool-aged (•) (n = 1) and school-aged (○) (n = 2) children with ADHD [49]. Solid line represents simulations for preschool-aged children and dashed line represents simulations for school-aged children; Panel B: Data as described for Panel A obtained after oral dosing with 5 mg MPH in preschool-aged (•) (n = 8) and school-aged (○) (n = 2) children [49]; Panel C: Data as described for Panel A obtained after oral dosing with 7.5 mg MPH in preschool-aged (•) (n = 4) and school-aged (○) (n = 1) children [49]; Panel D: Data as described for Panel A obtained after oral dosing with 10 mg MPH in preschool-aged (•) (n = 1) and school-aged (○) (n = 4) children [49]; Panel E: Data represent model simulated (lines) and observed plasma concentration of *d*-MPH after oral dosing with 5 mg (×), 10 mg (○), and 20 mg (•) MPH in boys with ADHD (n = 31) [50].

The calibrated children oral model was first tested against plasma *d-*MPH concentration time course data collected in children [49], [50] (Table S2). Figures 12A–D show model predictions and observations of plasma *d*-MPH concentrations over a period of 6 h in pre-school and school aged children administered a single oral dose of 2.5–10 mg MPH [49]. Time courses of plasma *d*-MPH concentrations were very well predicted across the dose range for both age groups, with the noted exception of school children treated with 2.5 mg of MPH (Figure 12A), for whom the measured plasma *d*-MPH levels were somewhat overestimated. Plasma *d*-MPH concentration time course data were also obtained in boys given a single oral dose of MPH at 5, 10 and 20 mg [50]. Model simulations in general agreed well with collected plasma *d*-MPH kinetic data over a period of 10 h for three groups, except that the model underestimated observations at 0.5 and 1 h for the 10 mg dose group but overestimated experimental data at 10 h for the 5 mg dose group within a factor of 2–4 (Figure 12E).

**Figure 13:**
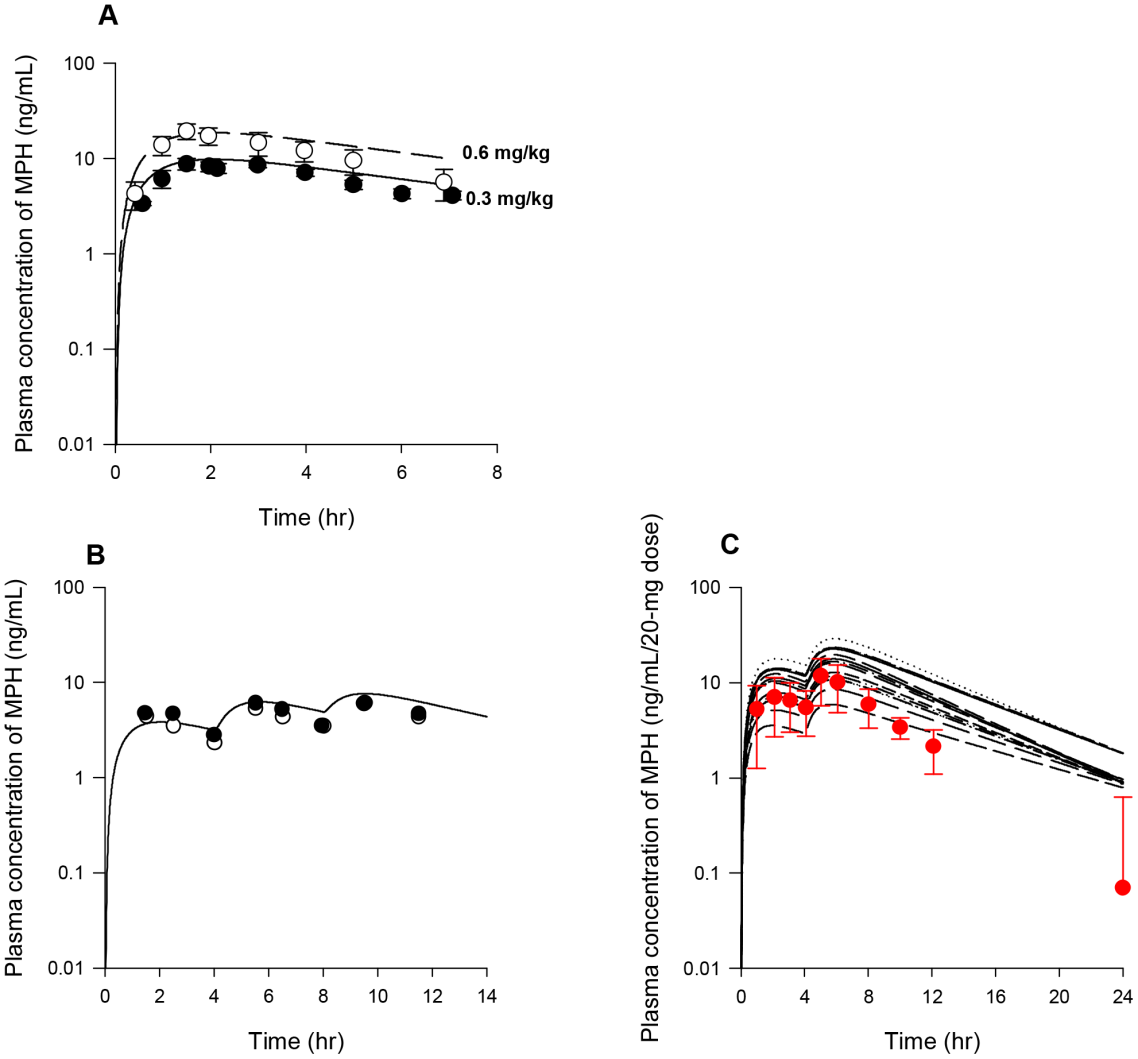
Plasma concentrations obtained after oral dosing of children with MPH. Panel A: Data represent model simulated (dashed line, 0.6 mg/kg; solid line, 0.3 mg/kg) and observed (circles) plasma concentration of MPH after oral dosing with 0.6 mg/kg (○) and 0.3 mg/kg (•) MPH in boys with ADD (n = 14) [47]; Panel B: Data represent model simulated (line) and observed plasma concentrations (○, fasting, •, normal) of MPH normalized to a dose of 5 mg after three repeated oral dosing with 5–15 mg MPH, taken 4 h apart, in children with ADHD (n = 14) [48]; Panel C: Data represent model simulated individual (lines) and observed (circles) plasma concentrations of MPH (•) normalized to a dose of 20 mg after two repeated dosing with 10–40 mg MPH, taken 4 h apart, in children with ADHD (n = 14) [51].

The calibrated children oral model was also evaluated against published data on plasma MPH concentrations collected in children after oral MPH administration [47], [48], [51] (Table S2). Single-dose kinetics of MPH in plasma was obtained in boys with ADD over a period of 7 h following oral administration of 0.342 and 0.651 mg/kg MPH [47]. Model simulations accurately tracked collected data (Figure 13A). Repeated-dose kinetics of MPH in plasma was obtained in children administered three repeated doses of 5–15 mg MPH taken 4 h apart, for which plasma MPH concentrations were normalized to a dose of 5 mg [48]. Simulation of plasma MPH kinetics over a period of 12 h was in excellent agreement with observed data (Figure 13B). In addition, the study of [51] also presented plasma MPH kinetic data following repeated oral dose of MPH at 10–40 mg taken 4 h apart, for which plasma MPH concentrations were normalized to a dose of 20 mg. Observations were in good agreement with model predictions, except that the simulated plasma levels of MPH were somewhat higher than measured levels for the later time points (from 8 to 24 h) (Figure 13C).

### Model Evaluation: Juvenile Monkeys

**Figure 14:**
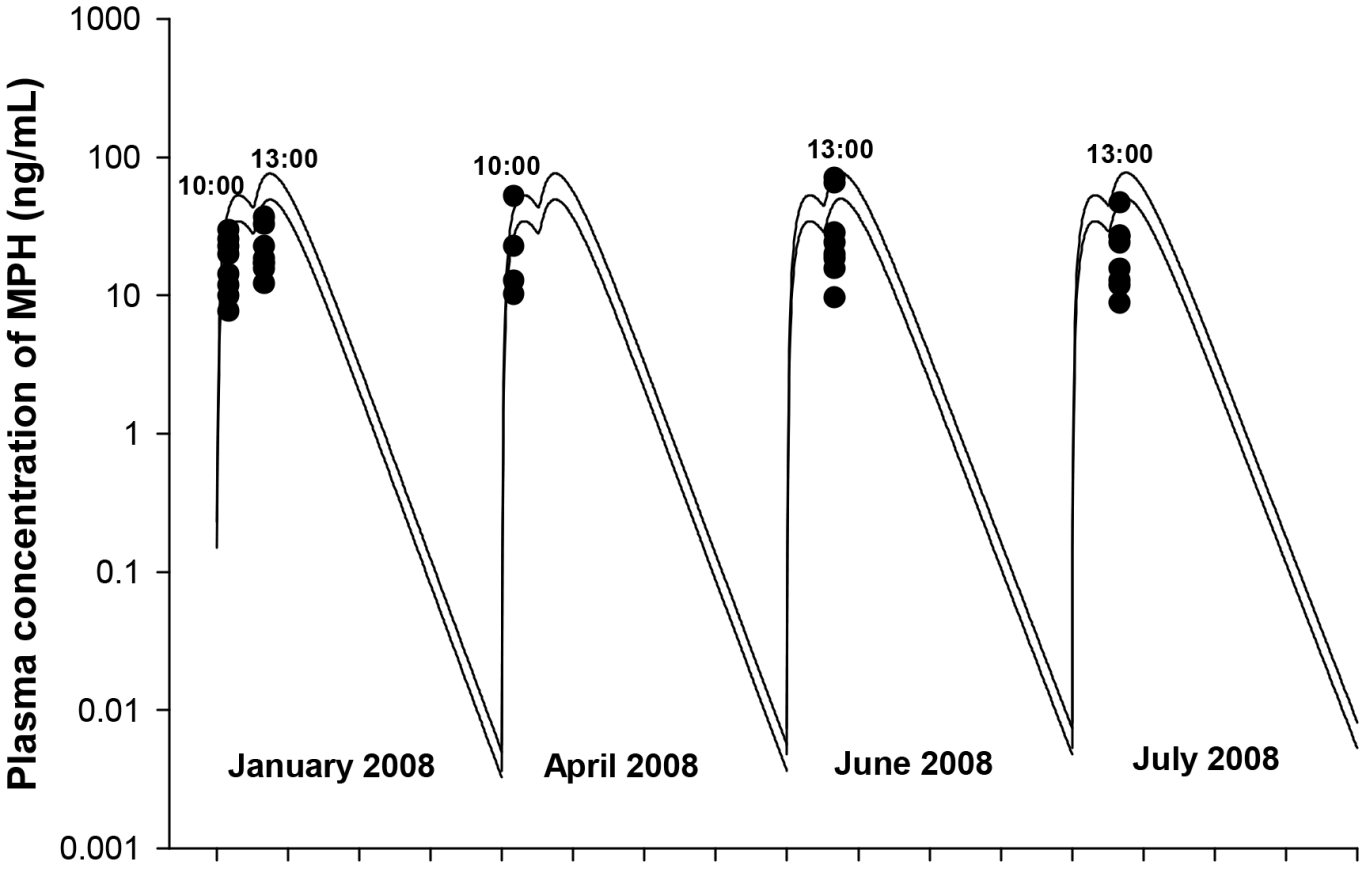
Plasma concentrations obtained after repeated oral dosing of juvenile monkeys with MPH. Data represent model simulated (lines) and observed (circles) individual plasma concentrations of MPH at 10∶00 and 13∶00 after daily oral dosing with either 10.7 mg/kg (lower line) or 16.5 mg/kg (upper line) MPH at 9∶00 and 12∶00 (n = 8) [27].

Figure 14 shows model predictions and observations of MPH plasma concentrations in juvenile male rhesus monkeys repeatedly administered 10.7 (8.89–13.1) mg/kg or 16.5 (15.5–18.7) mg/kg of MPH, twice daily, 3 h apart (at 9∶00 and 12∶00) [27]. When using the calibrated young monkey oral model, model predictions of MPH plasma concentrations collected one hour after oral dosing, at 10∶00 and 13∶00, were in general agreement with observations (Figure 14).

### Assessment of Model Prediction Performance

Model prediction performance of plasma MPH or *d-* and *l-*MPH levels was assessed using RMSE for data sets in children reported by [7], [46]–[51] and for data sets in juvenile monkeys reported in this paper (NCTR data) and from Johns Hopkins University [27].

The RMSE values for data sets used for children oral model calibration [7], [46] ranged from 0.3 to 2.0, with a mean of 1.0. The calibrated model demonstrated similar performance when tested against other data sets [47]–[51], yielding RMSE values ranged from 1.11–4.88, with a mean of 2.4, of which, the RMSE value (24.85) estimated for the study of [51] was excluded as an outlier. The RMSE values for data sets used for juvenile monkey oral model calibration (NCTR data) ranged from 3.4 to 6.0, with a mean of 4.1 for the 2.5 mg/kg dose group; and for the 12.5 mg/kg dose group, ranged from 16–106, with a mean of 61.5. Similar prediction performance as the high dose group (12.5 mg/kg) was observed for juvenile monkey oral data sets (10.7 and 16.5 mg/kg) from Johns Hopkins University [27] used for model evaluation, with average RMSE value of 31.5 (20–40), suggesting that the model performs better for the low dose group (2.5 mg/kg) for the juvenile monkey oral model. Comparable prediction performance of the juvenile monkey oral model for the 2.5 mg/kg dose (mean RMSE = 4.1) and the children oral model (mean RMSE = 2.1, for data sets used for children oral model calibration and evaluation [7], [46]–[50]) provides confidence for interspecies extrapolation of toxicity findings from the 2.5 mg/kg juvenile moneys dose group to children.

### Interspecies Extrapolation (Monkey to Human)

The developed PBPK model was utilized to derive oral HEDs for boys 6 and 15 years of age to produce equivalent internal doses for MPH associated with observed pubertal delays in juvenile monkeys chronically exposed to 2.5 mg/kg MPH [26].

For juvenile male rhesus monkeys given repeated oral doses of 2.5 mg/kg MPH, model simulated peak concentration (Cmax) and adjusted daily AUC for MPH at steady state were 10.4 ng/mL and 50.3 ng/mL*h per day (Table S3). To achieve equivalent Cmax at steady state as that in juvenile monkeys, model derived HEDs in boys 6 and 15 years of age were 0.183 mg/kg (3.9 mg) and 0.261 mg/kg (15.5 mg); and for the dosimetry of daily AUC, 0.084 mg/kg (1.8 mg) and 0.114 mg/kg (6.8 mg). These derived HEDs (0.084–0.261 mg/kg) are below the recommended MPH doses for children (0.3–0.8 mg/kg) by the American Academy of Pediatrics.

Consistently, model estimated internal dose metrics (Cmax and daily AUC) for boys either 6 or 15 years of age receiving recommended MPH doses (0.3–0.8 mg/kg) [90] are greater than those associated with observed pubertal delays in juvenile monkeys (Table S3). Following repeated daily oral administration of 0.3 mg/kg MPH, model simulated Cmax and daily AUC at steady state were 17.2 ng/mL and 180.4 ng/mL*h per day for boys 6 years of age; and for boys 15 years of age, 12.0 ng/mL and 131.9 ng/mL*h per day. Following repeated oral administration of 0.8 mg/kg MPH for a week, model simulated Cmax and daily AUC were 45.8 ng/mL and 481.4 ng/mL*h per day for boys 6 years of age; and for boys 15 years of age, 31.9 ng/mL and 351.8 ng/mL*h per day.

### Sensitivity Analysis

Table 5 presents model parameters determined to be sensitive with absolute NSC values greater than 0.1 using the time course of MPH plasma concentrations over a period of 24 h as the model output. A similar sensitivity pattern was observed across species and age, with some exceptions. For example, blood flows to the fat and the slowly perfused tissues (QFC and QSC) were found to be sensitive for monkeys, but not for humans. In addition, parameters responsible for the hepatic hydrolysis of *l*-MPH (Kmliverl and VmaxliverlC) were sensitive for young humans and monkeys, but not for adults. Of note, some parameters, e.g. cardiac output (QCC) and hepatic hydrolysis of *d*-MPH (Kmliverd and Vmaxliverd), appear to impact the plasma concentrations of MPH to a greater extent with absolute NSC values larger than 1.

**Table 5:** Sensitive model parameters.

	Physiological Parameters	Partition Coefficients	Chemical specific model parameters
**Adult Humans**	QCC, QLC, QRC, BW,VPC, VFC, VLC, VRC, VSC	Pfat, Prich, Pslow, Pliver	**Kmliverd**, **VmaxliverdC**, KmetdC, GEdC, K3dC, K5dC, GElC, K3lC, K5lC
**Children**	**QCC**, QLC, QRC,**BW,** VPC, VFC, VLC, VRC,VSC, **Age**	Pfat, Prich, Pslow,Pliver, Pbrain	**Kmliverd**, **VmaxliverdC,** KmetdC, GEdC, K3dC, K5dC, GElC,K3lC, K5lC, Kmliverl, VmaxliverlC
**Adult Monkeys**	**QCC, QLC**, QRC, QFC, QSC, **BW,** VPC, VLC,VRC, **VSC**	Pfat, **Prich**, **Pslow**,Pliver, Pbrain	**Kmliverd**, **VmaxliverdC**, **KmetdC**, GEdC, K3dC, K5dC, GElC, K3lC, K5lC
**Juvenile Monkeys**	**QCC, QLC, QRC,QFC,** QHC, QSC, **BW,** VPC,**VFC**, VLC, VRC, **VSC**	**Pfat,** **Prich, Pslow**,Pliver, Pbrain	Kmliverd, VmaxliverdC, KmetdC, GEdC, **K3dC**, GElC, K3lC, Kmliverl,VmaxliverlC, KmetlC

Parameters with absolute NSC values greater than 1 are highlighted in bold.

With the exception of kinetic parameters associated with *l*-MPH found to be nonsensitive, the same sensitivity pattern was observed with regards to the time course of plasma *d*-MPH concentrations over a period of 24 h. This is consistent with the finding that plasma concentrations of *l*-MPH were negligible and plasma concentrations of the *d*-enantiomer approximated those of the racemic MPH.

## Discussion

A PBPK model was constructed for the first time to describe the kinetic behavior of MPH and its primary metabolite RA in young and adult humans and non-human primates. The availability of plasma concentration time courses of *d*- and *l*-MPH [4], [29]–[31] and urinary excretion profiles of *d*- and *l*-RA in adult humans [28], coupled with the characterization of the hydrolysis of MPH enantiomers using the recombinant human CES1A1 enzyme [16], provide confidence in the appropriate estimation of enantiomer-specific kinetic parameters for both MPH and RA. As a result, the PBPK model was first calibrated in adult humans, and then extrapolated to children and young and adult monkeys with incorporation of potential species- and age-dependent differences in MPH disposition.

With some exceptions, many model predictions are off by a factor of 2–3 compared to experimental data. As recommended in WHO PBPK guidance document [93], “In PBPK modelling, predictions that are, on average, within a factor of 2 of the experimental data have frequently been considered adequate. When the training (or parameter estimation) data set and evaluation data set are obtained in different experimental animals/human subjects, as in most PBPK modelling activities, the resulting simulations are not anticipated to fit the PK data perfectly at all time points”. Also, the evaluation of model value should consider “biological basis and reliability of dose metric predictions” in addition to “closeness to data”. Hence, the performance of the current MPH PBPK model is judged to be adequate for practical application.

### Model Development: Humans

To describe MPH hydrolysis in the liver, Michaelis affinity constants, Kmliverd and Kmliverl, were from literature, while VmaxliverdC and VmaxliverlC constants were determined by parameter fitting. In vitro intrinsic liver clearance values, calculated as Vmaxliver/Kmliver, for a 70 kg person, are estimated to be 0.48 L/h/kg bw for *d*-MPH and 3.06 L/h/kg bw for *l*-MPH. These values fall between those observed for rapidly cleared drugs, e.g. deltamethrin (9.7 L/h/kg bw) [94] and oseltamivir (8.4 L/h/kg bw) [95], [96], which are also human carboxylesterase I substrates, and the slowly cleared drug, e.g amphetamine (0.004 L/h/kg bw) [97], which is structurally close to MPH. Hence, it appears that parameter values for VmaxliverC obtained by fitting the kinetic data are within the physiological range. However, since many parameters required for a fully mechanistic PBPK model are not available in literature and were determined by parameter fitting, some of them may lack physiological significance. To reduce the uncertainty and strengthen the robustness of the model, additional studies are needed to estimate model parameters.

With hepatic metabolic parameters derived from intravenous dosing of adult humans [4], describing kinetic behaviors of MPH following oral administration [24], [29]–[33] became a challenge. The model consistently overestimated plasma MPH levels after oral administration, even with a low oral uptake constant. To account for the relatively low plasma concentrations of MPH observed in orally dosed adult humans, we hypothesized that pre-systemic metabolism of MPH also occurred in the small intestine, via both hydrolytic [73]–[75] and oxidative [76] metabolic pathways. However, further studies are needed to better understand the fate of MPH in the GI tract and to more reasonably estimate related model parameters. MPH emptied from the stomach into the small intestine lumen was assumed to be available immediately within enterocytes, where MPH was either taken up into the portal blood circulation or metabolized. The calibrated adult human model for MPH suggested that approximately 85% of MPH administered orally is metabolized in the small intestine of adult humans, with the remaining taken up into the liver.

For children, another issue arose. The lack of iv dosing data made it difficult for reasonable estimation of kinetic model parameters because oral route of administration confounds the kinetic interpretations. Thus, the calibrated adult human oral model was extrapolated to describe the plasma kinetics of orally administered MPH in children. Scaling of constants describing hepatic and gut metabolism as well as oral uptake of MPH, determined by optimization in adult humans, provided a sufficient description of plasma *d-*MPH levels in children. However, recalibration of the gut metabolism constant for *l-*MPH (K5lC, 1/h/kg^0.75^) was needed to accurately track the kinetic behavior of *l-*MPH in children. The adult value of K5lC was decreased by 13 fold to account for the slower systemic clearance of *l-*MPH in children. Plasma RA concentrations in children [19] were predicted using scaled adult urinary excretion (clearance) constants.

Sensitivity analysis indicated that model parameters representing hepatic and intestinal metabolism of MPH appeared to significantly impact model predictions of plasma MPH concentrations in humans, both adults and children. To obtain more reasonable estimates of these parameter values, studies using *in vitro* preparations are needed to fully investigate the age-dependent metabolism of MPH enantiomers (*d*- and *l*-MPH) in the liver and the small intestine. With these new data, it may be possible to derive a scaling approach to describe the maturation of liver and gut metabolism of MPH. Additionally, in the current model, the resultant metabolite RA was assumed to be taken up immediately into the systemic circulation, and hence the rate of RA formation after oral administration equals the rate of MPH hydrolysis in the liver and the small intestine. Research is necessary to examine the transport mechanisms of RA in the liver and the small intestine. Also, pharmacokinetic studies in children following iv administration of MPH with simultaneous quantification of MPH and RA enantiomers would be of critical importance for reasonable characterization of hepatic metabolism of MPH and systemic clearance of RA in children.

### Model Development: Monkeys

For monkeys, no *in vitro* metabolism studies were available for the derivation of hepatic metabolic constants and those limited kinetic studies from NCTR and Wargin et al. [24] have been restricted to non-enantiospecific analytic approaches (i.e. reporting only pooled *d*- and *l*-MPH concentrations). Further, the metabolism and excretion pathways of MPH have not been well described in monkeys. The determination of enantiomer-specific model parameters for monkeys became challenging. As such, the development of the monkey MPH PBPK model relied primarily on interspecies and intraspecies extrapolation using allometric scaling, with new pharmacokinetic data collected at NCTR and from Wargin et al. [24] as the primary sources for model calibration.

The scaled model parameters describing hepatic metabolism of *d-* and *l-*MPH calibrated in adult humans by iv administration in general predicted plasma kinetics of *d-* and *l-*MPH in adult monkeys after iv dosing. Correspondingly, scaling of adult human urinary excretion (clearance) constants described plasma *d-* and *l-*RA kinetics for adult monkeys. However, when extrapolating the adult human oral model to adult monkeys, recalibration of gut metabolism constants for *d-* and *l-*MPH was needed to account for the potential greater gut metabolic capacity in monkeys than humans [75], [86], [87], [92]. With MPH-specific model parameters recalibrated for adult monkeys after oral dosing, the scaled constants describing systemic excretion of *d-* and *l-*RA from adult humans worked well for the prediction of plasma *d-* and *l-*RA kinetics for adult monkeys.

Next, the calibrated adult monkey iv and oral models were extrapolated to describe the plasma kinetics of MPH in juvenile monkeys given iv and oral doses. However, these intraspecies extrapolations were not successful and recalibration was needed. For iv dosing, plasma MPH is cleared more rapidly in young monkeys [24] compared to adult monkeys (NCTR data) and larger hepatic metabolic constants for *d*- and *l*-MPH were required. Scaled hepatic hydrolysis terms (Vmaxliverd and Vmaxliverl) and hepatic oxidation terms (Kmetd and Kmetl) are estimated to be 9.9E^5^ µg/h, 2.0E^6^µg/h, 198 L/h and 198 L/h for juvenile monkeys of 2.5 years old [24]; while for adult monkeys, these values are estimated to be 2.1E^5^ µg/h, 4.9E^5^ µg/h, 3.83L/h, and 3.83 L/h, respectively. In addition, model simulations suggested that in young monkeys microsomal oxidation represents a major route (approximately 68%) for MPH metabolism, similar to those reported for rats and dogs [18], [21]; while in adult monkeys, the administered dose is thought to be predominantly metabolized by hydrolysis (approximately 80%), similar to what is found in humans [18]. With these MPH-specific model parameters recalibrated, scaling of adult monkey urinary excretion constants for RA worked well for the description of plasma RA kinetics after iv dosing in juvenile monkeys.

For oral dosing in juvenile monkeys, with hepatic metabolic constants recalibrated from the juvenile monkey iv data [24], scaling of adult monkey oral uptake constants for *d-* and *l-*MPH worked well for the prediction of plasma *d-* and *l-*MPH kinetics in juvenile monkeys. However no gut metabolism was assumed. Subsequently, plasma *d-* and *l-*RA kinetics in juvenile monkeys after oral dosing was successfully described using scaled systemic clearance terms for *d-* and *l-*RA determined in adult monkeys.

Due to lack of knowledge, the calibration of the monkey MPH model is an exploratory evaluation of MPH pharmacokinetics in monkeys. The metabolic pathways of MPH and systemic clearance of RA in monkeys were assumed to be similar to those identified in humans, and model parameter values were primarily fit for purpose but without adequate empirical evidence. This is a major concern of the current model. As such, there is more uncertainty with regard to the estimation of model parameters for the monkey model compared with the human model. To address these uncertainties, further research using both *in vitro* and *in vivo* systems is needed to determine if monkeys and humans process MPH in a similar fashion and to provide evidence for reasonable determination of kinetic model parameter values in monkeys. Also, similar to humans, more studies need to be conducted to explore the effect of age on the metabolism and excretion of intravenously and orally dosed MPH in monkeys. In addition, sensitivity analysis suggested that model outputs were also sensitive to physiological parameters, e.g. cardiac output (QCC), blood flow to the liver (QLC), and tissue volumes of the liver and plasma (VLC and VPC). However, such physiological information is not available for young monkeys, which were set to adult monkey values in the current model. Characterization of physiological parameters for young monkeys is required to strengthen the model.

### Model Improvement

In addition to the issues discussed above that need to be addressed to increase model prediction performance, the current model can be further improved in the following respects.

First, although immediate-release MPH has been established as the “gold standard” for the treatment of ADHD, with the rapid introduction of novel extended-release MPH dosage forms into the market, it is of clinical relevance to also describe the pharmacokinetic behaviors of extended-release MPH formulations. Compared with immediate-release MPH, which is rapidly absorbed from the intestine, the extended absorption profile of extended-release MPH dosage forms are primarily controlled by programmed dissolution and release kinetics [12]. By taking into account the characteristics of the extended-release formulations and their interactions with the gastrointestinal tract, the current model can be expanded to describe the pharmacokinetics of extended-release MPH formulations.

Second, the advances in the understanding of pharmacological [42] and toxicological mechanisms underlying the action of MPH offer the possibility of incorporating the mechanistic component (pharmacodynamic, PD) into the current PBPK model. The establishment of the PBPK/PD model will allow for simultaneous estimation of the internal dose metrics and associated biological effects of MPH, and may provide insights into the causes of individual variability in response to MPH treatment from both pharmacokinetic and pharmacodynamic perspectives.

Third, large interpatient variability has been reported for pharmacokinetics and clinical response of MPH and dosage must be titrated for optimal effects [57], [98]. With the incorporation of statistical simulation techniques (i.e. Monte Carlo simulations) to account for the probability distribution of physiological and biochemical characteristics, the PBPK/PD model can better address such large interindividual differences. In addition, pharmacogenetic studies have reported the impact of polymorphisms of MPH targets (catecholamine candidate genes) [99] on individual MPH-responses. Also, ethnic differences have been observed for the hCES1 enzyme [100] and functional polymorphisms (mutations) of the hCES1 gene with reduced enzyme activity have been identified [101], [102]. Further efforts can be made to develop a mechanistic covariate model by integrating polymorphism into the PBPK/PD model, which can provide clues for individualized MPH regimens based on genetic information.

Fourth, in the current model, plasma dose metrics of MPH were employed for species extrapolation. Though kinetics of MPH in plasma and tissues (e.g. heart and brain) were generally in parallel, as shown in rats [58]–[60], dose metrics of target tissues might be more representative of risk. However, although a direct effect of MPH on the testis or an indirect effect at a site or sites in the pituitary or hypothalamus has been implicated [26], the underlying mechanisms associated with MPH induced changes in testosterone levels have not yet been unraveled. In addition, though MPH metabolites are pharmacologically inactive, with the exception of *p-*hydroxymethylphenidate [17], [18], to our best knowledge, no studies have been conducted to evaluate the toxicity of MPH metabolites. Hence, further research is required to identify the exact cellular and molecular processes involved in the potential toxicity caused by MPH. Identified target tissues and the mechanisms involved can be integrated into our PBPK model, allowing a more accurate extrapolation of MPH effects across species and age.

Other confounding issues also need further study. Some studies reported that food may affect the absorption of either immediate-release or extended-release MPH [39], while others did not [103]. Also sexual dimorphism in MPH pharmacokinetics has been reported: women appear to require larger mg/kg doses to achieve the same MPH plasma concentration as men, which might be attributed to the more extensive first pass metabolism of MPH in women [12], [104]. However such a sex difference was not noticed for children with ADHD [98]. With further studies performed to verify the impact of food and sex on the disposition of MPH, related kinetic parameters might be adjusted to account for such effects.

### Interspecies Extrapolation

With the incorporation of known variations in physiological factors between experimental animals and humans, PBPK model based interspecies extrapolation has become a useful tool to quantitatively evaluate internal doses of a chemical or a drug across species. The success of default scaling cross species seems age- and route- dependent. Scaling of the adult human model parameters to adult monkeys works well for predicting MPH pharmacokinetics after intravenous dosing; while for oral dosing, recalibration of gut metabolism constants derived from the adult human oral model is needed. Contrarily, if extrapolating the children oral model to juvenile monkeys, both hepatic and gut metabolism constants need to be recalibrated. The necessity to recalibrate model parameters obtained by default scaling for interspecies extrapolation implies latent cross species variations in the pharmacokinetics of MPH.

To better understand the disposition of MPH across species and age, daily AUC values of MPH at steady state following repeated daily oral dosing of MPH at 0.3 mg/kg, twice a day with 4 h apart, were assessed for juvenile and adult monkeys and humans. Consistent with the finding that juvenile monkeys require larger oral MPH doses to achieve similar serum levels of MPH in adult monkeys and humans as well as children [22], [23], [26], , the daily AUC value (7.9 ng/mL*h per day) of MPH in juvenile monkeys is far below those in adult monkeys (25.1 ng/mL*h per day), adult humans (107.1 ng/mL*h per day), and boys of 6 (179.2 ng/mL*h per day) and 15 (130.6 ng/mL*h per day) years old. Juvenile monkeys appear to metabolize MPH more rapidly than adult monkeys and humans as well as children, which is accounted for by using larger hepatic metabolic constants for juvenile monkeys in the model. The lower systemic exposure of MPH in adult monkeys compared with adult humans and children could be explained by the potential greater extent of first-pass metabolism occurred in the gut of adult monkeys [75], [86], [87]. In addition, children were found to have higher internal dose metrics compared with adult humans: the most likely explanation might be that children have lower gut metabolism capacity for the *l*-MPH, as assumed in the model.

Next, PBPK model based interspecies extrapolation was employed for human risk assessment of MPH. Due to the limitations for direct evaluation of the toxicity of environmental chemicals or drugs in humans, species extrapolation of toxicity data from experimental animals has been commonly used to predict responses in humans despite differences may exist in how humans and experimental animals respond to chemicals. In the current model, with the observed pubertal delay in young monkeys administered 2.5 mg/kg MPH as the endpoint of interest, model derived HEDs (0.183 mg/kg and 0.261 mg/kg for boys 6 and 15 years of age using Cmax as the dosimetry; and 0.084 and 0.114 mg/kg for daily AUC dosimetry) are below the recommended MPH doses for children (0.3–0.8 mg/kg) by the American Academy of Pediatrics. Of note, consistent with the finding that chronic MPH exposure resulted in temporary intervention of serum testosterone concentrations in juvenile male rhesus monkeys [26], [27], higher salivary testosterone levels and atypically flat circadian rhythms in salivary testosterone have been reported in children receiving MPH [25]. Since the impairment of pubertal development noticed in monkeys was only transitory, the concerns about the clinical use of MPH in pre-pubescent children may be somewhat relieved. However, given the widespread use of MPH and related amphetamines in the pediatric population, more studies in both animal models and humans need to be conducted to fully describe the effects of MPH, particularly those associated with chronic treatment.

## Supporting Information

Figure S1 **Plasma concentrations obtained after repeated oral dosing of juvenile male monkeys with MPH (NCTR data).** Data represent model simulated (lines) and observed (circles) individual plasma concentrations of MPH (•) after repeated oral dosing with 2.5 mg/kg MPH (n = 1–4 at each time point) across the study. MPH was administered twice a day, five days a week (Monday to Friday) and kinetic studies were performed from Monday to Thursday. On the day of blood collection, MPH was administered only once in the morning (solid lines). Dashed lines represent plasma concentration time courses of MPH and RA under repeated dosing schedules.
(TIF)

Figure S2 **Plasma concentrations obtained after repeated oral dosing of juvenile male monkeys with MPH (NCTR data).** Data represent model simulated (lines) and observed (circles) individual plasma concentrations of RA (•) after repeated oral dosing with 2.5 mg/kg MPH (n = 1–4 at each time point) across the study. Measurements of plasma RA concentrations at pre-dose (approximately within 30 min of dosing) were combined with those at 24 h from previous dose. MPH was administered twice a day, five days a week (Monday to Friday) and kinetic studies were performed from Monday to Thursday. On the day of blood collection, MPH was administered only once in the morning (solid lines). Dashed lines represent plasma concentration time courses of MPH and RA under repeated dosing schedules.
(TIF)

Figure S3 **Plasma concentrations obtained after repeated oral dosing of juvenile male monkeys with MPH (NCTR data).** Data represent model simulated (lines) and observed (circles) individual plasma concentrations of MPH (•) after repeated oral dosing with 12.5 mg/kg MPH (n = 1–4 at each time point) across the study. MPH was administered twice a day, five days a week (Monday to Friday) and kinetic studies were performed from Monday to Thursday. On the day of blood collection, MPH was administered only once in the morning (solid lines). Dashed lines represent plasma concentration time courses of MPH and RA under repeated dosing schedules.
(TIF)

Figure S4 **Plasma concentrations obtained after repeated oral dosing of juvenile male monkeys with MPH (NCTR data).** Data represent model simulated (lines) and observed (circles) individual plasma concentrations of RA (•) after repeated oral dosing with 12.5 mg/kg MPH (n = 1–4 at each time point) across the study. Measurements of plasma RA concentrations at pre-dose (approximately within 30 min of dosing) were combined with those at 24 h from previous dose. MPH was administered twice a day, five days a week (Monday to Friday) and kinetic studies were performed from Monday to Thursday. On the day of blood collection, MPH was administered only once in the morning (solid lines). Dashed lines represent plasma concentration time courses of MPH and RA under repeated dosing schedules.
(TIF)

Figure S5 **Plasma concentrations obtained after oral dosing of healthy adult humans with MPH.** Panel A: Data represent model simulated (lines) and observed individual (circles) plasma concentrations of *d*-MPH after oral dosing with 20 mg MPH (n = 4) [40]; Panel B: Data represent model simulated (line) and observed (circles) plasma concentrations of MPH after two repeated dosing with 10 mg MPH, taken 5 h apart (n = 18) [43]; Panel C: Data represent model simulated (line) and observed (circles) plasma concentrations of *d*-MPH after oral dosing with 40 mg of MPH (n = 24) [39]; Panel D: Data as described for Panel A obtained after oral dosing with 40 mg MPH (n = 6) [42]; Panel E: Data represent model simulated (lines) and observed plasma concentrations of *d*-MPH (•) and *d*-RA(▴) after oral dosing with 20 mg *d*-MPH (n = 15) [41].
(TIF)

Figure S6 **Plasma concentrations obtained after oral dosing of adult humans with MPH.** Panel A: Data represent model simulated (lines) and observed plasma concentrations of d-MPH (•), l-MPH (○), d-RA (▴) and l-RA(Δ) after oral dosing with 10 mg MPH (n = 1) [38]; Panel B: Data as described for Panel A obtained after oral dosing with 20 mg MPH (n = 1) [38]; Panel C: Data as described for Panel A obtained after oral dosing with 30 mg MPH (n = 1) [38]; Panel D: Data as described for Panel A obtained after oral dosing with 40 mg.
(TIF)

Table S1 **Immediate release MPH pharmacokinetic studies used for model calibration and evaluation for healthy adult male and female humans.**
(DOC)

Table S2 **Immediate release MPH pharmacokinetic studies used for model calibration and evaluation for male and female children with ADHD and ADD.**
(DOC)

Table S3 **Human equivalent dose (HED) calculations for MPH in boys either 6 or 15 years of age based on juvenile male rhesus monkey toxicity and pharmacokinetic studies with MPH.** For juvenile monkeys, experimental daily AUC was calculated as total AUC over one week divided by 5 days and adjusted daily AUC used for HED calculations was calculated as total AUC over one week divided by 7 days; for boys, daily AUC was calculated as (total AUC over one week − total AUC from Money to Wednesday) divided by 4 days.
(DOC)

Text S1 **Additional pharmacokinetic studies in humans.**
(DOC)

Text S2 **Methylphenidate PBPK model code.**
(TXT)

Text S3 **PBPK model code for physiological parameter estimation in children.**
(TXT)
