# Supplementary figures and images for: Development of a Physiologically Based Model to Describe the Pharmacokinetics of Methylphenidate in Juvenile and Adult Humans and Nonhuman Primates
Source: PLoS One. 2014 Sep 3;9(9):e106101. doi: 10.1371/journal.pone.0106101 (PMC4153582; doi:10.1371/journal.pone.0106101)

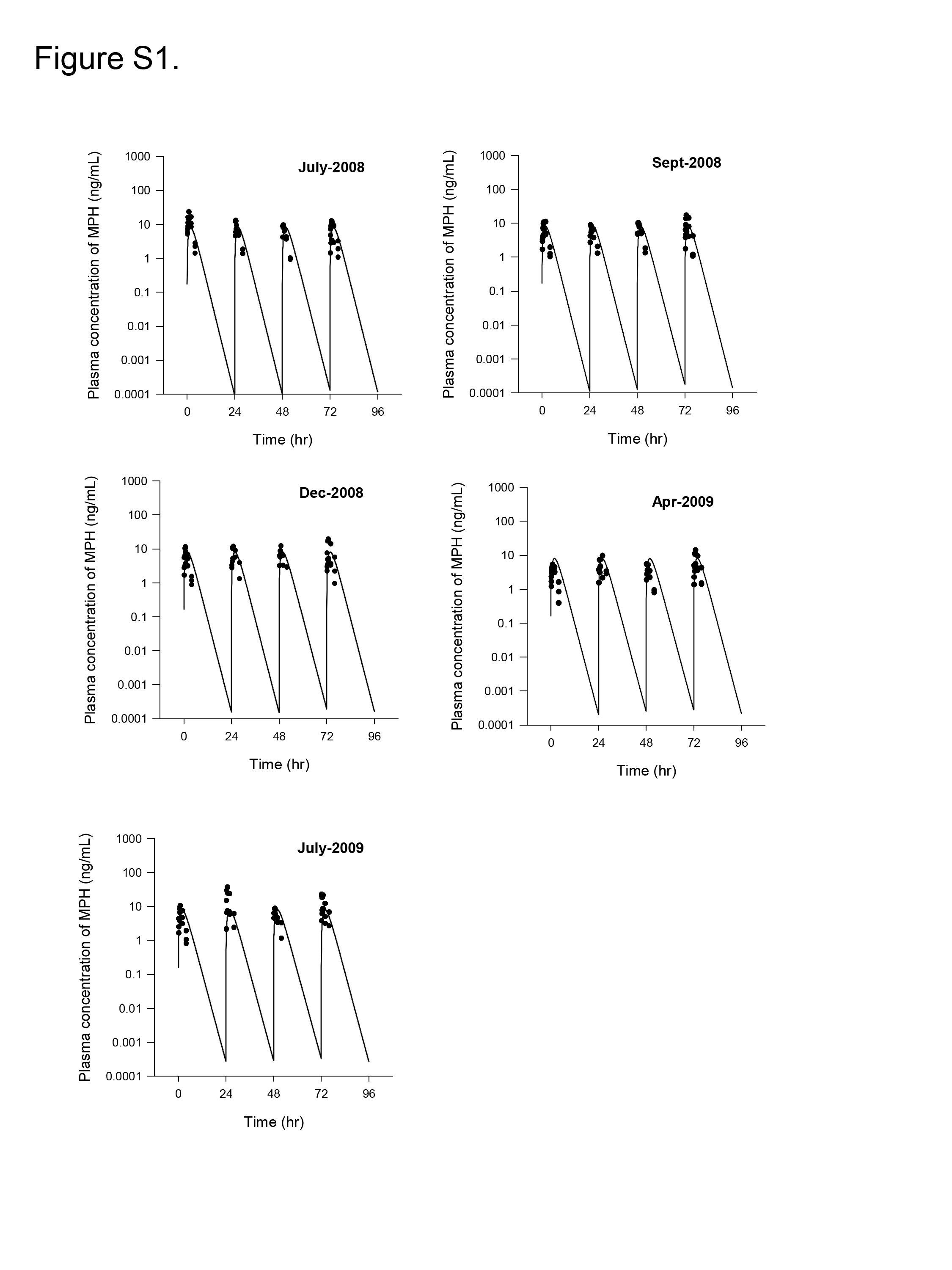

Supplement: Figure S1 — Plasma concentrations obtained after repeated oral dosing of juvenile male monkeys with MPH (NCTR data). Data represent model simulated (lines) and observed (circles) individual plasma concentrations of MPH (•) after repeated oral dosing with 2.5 mg/kg MPH (n = 1–4 at each time point) across the study. MPH was administered twice a day, five days a week (Monday to Friday) and kinetic studies were performed from Monday to Thursday. On the day of blood collection, MPH was administered only once in the morning (solid lines). Dashed lines represent plasma concentration time courses of MPH and RA under repeated dosing schedules. (TIF) [file pone.0106101.s001.tif]

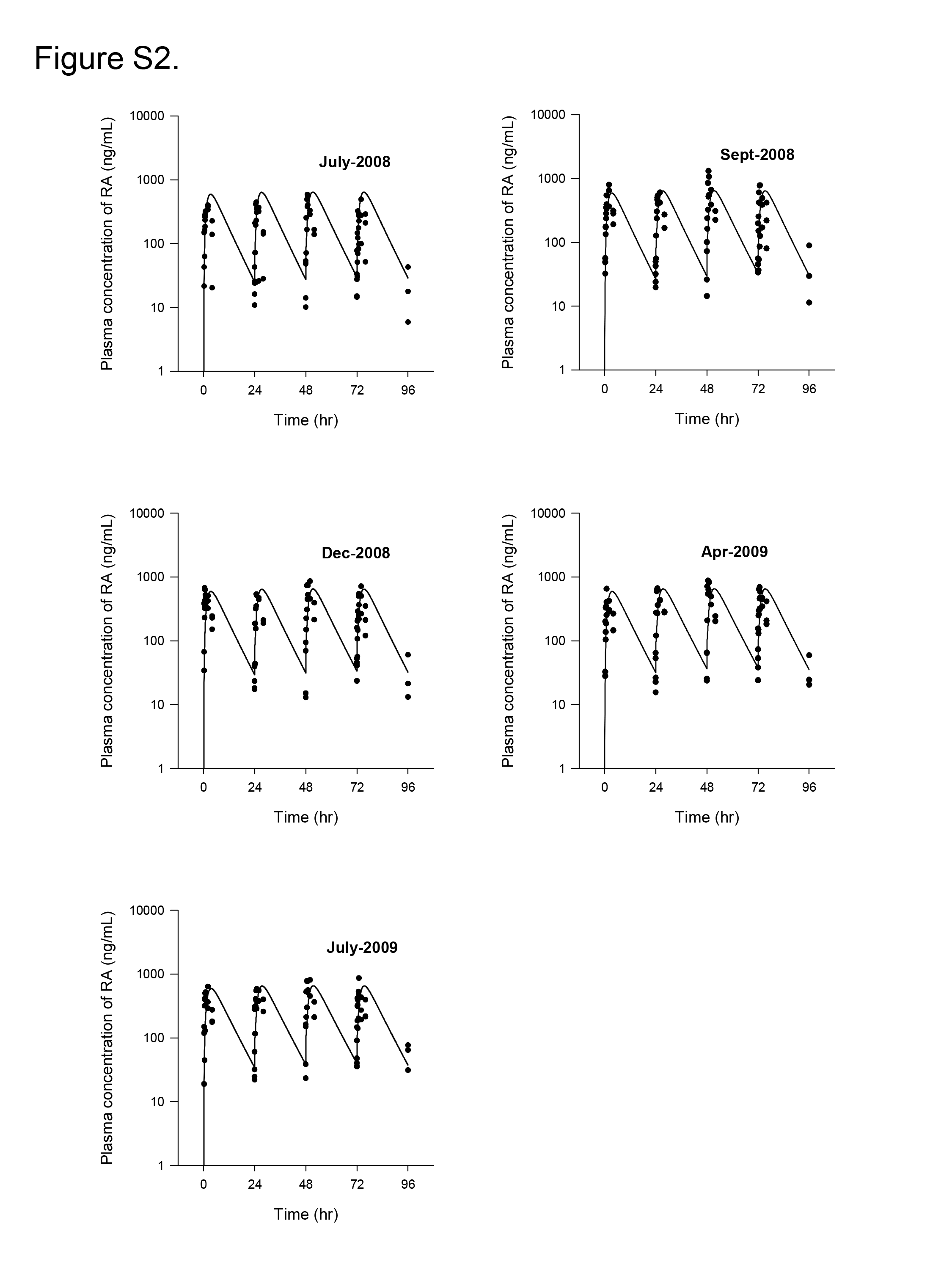

Supplement: Figure S2 — Plasma concentrations obtained after repeated oral dosing of juvenile male monkeys with MPH (NCTR data). Data represent model simulated (lines) and observed (circles) individual plasma concentrations of RA (•) after repeated oral dosing with 2.5 mg/kg MPH (n = 1–4 at each time point) across the study. Measurements of plasma RA concentrations at pre-dose (approximately within 30 min of dosing) were combined with those at 24 h from previous dose. MPH was administered twice a day, five days a week (Monday to Friday) and kinetic studies were performed from Monday to Thursday. On the day of blood collection, MPH was administered only once in the morning (solid lines). Dashed lines represent plasma concentration time courses of MPH and RA under repeated dosing schedules. (TIF) [file pone.0106101.s002.tif]

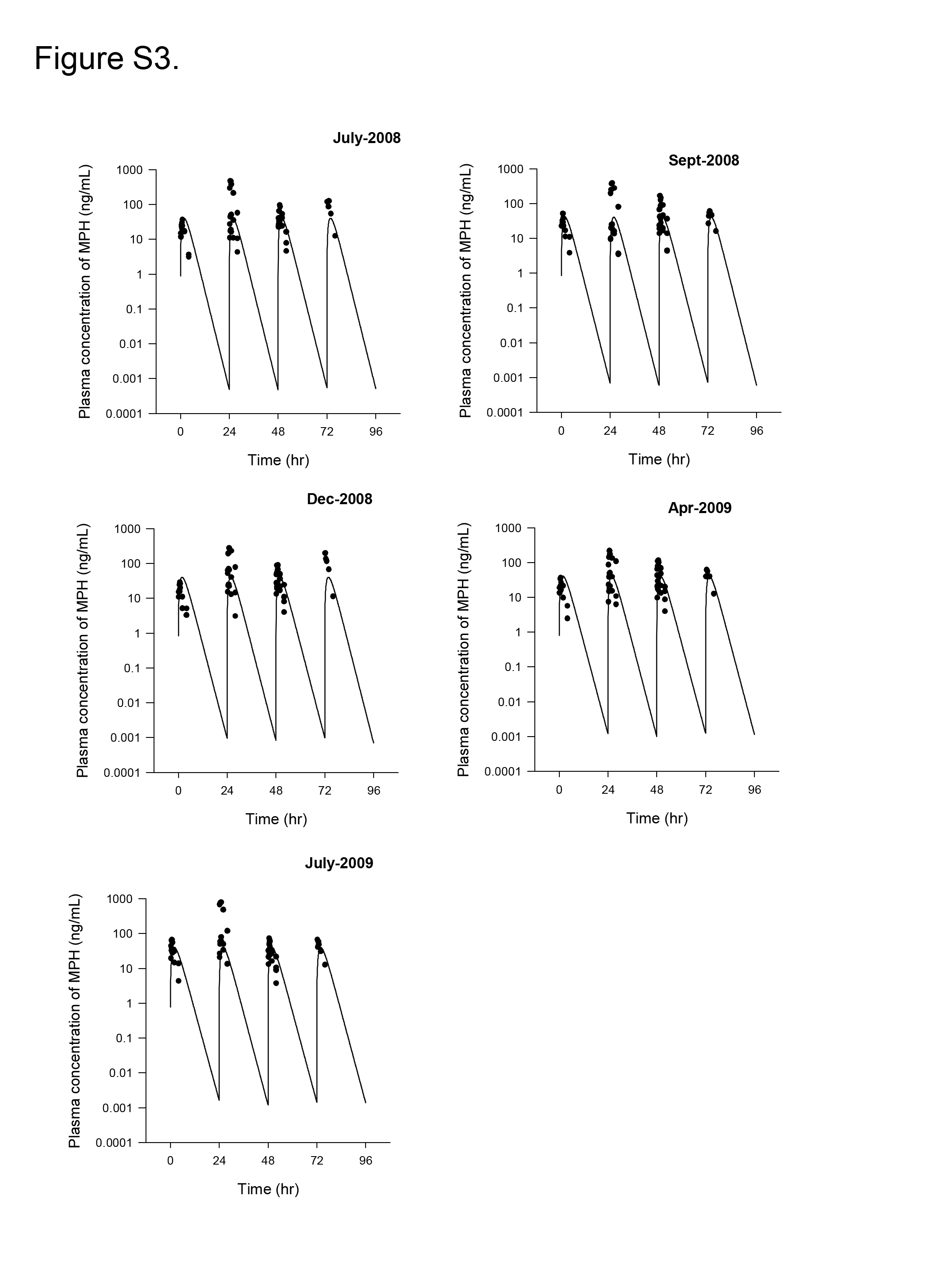

Supplement: Figure S3 — Plasma concentrations obtained after repeated oral dosing of juvenile male monkeys with MPH (NCTR data). Data represent model simulated (lines) and observed (circles) individual plasma concentrations of MPH (•) after repeated oral dosing with 12.5 mg/kg MPH (n = 1–4 at each time point) across the study. MPH was administered twice a day, five days a week (Monday to Friday) and kinetic studies were performed from Monday to Thursday. On the day of blood collection, MPH was administered only once in the morning (solid lines). Dashed lines represent plasma concentration time courses of MPH and RA under repeated dosing schedules. (TIF) [file pone.0106101.s003.tif]

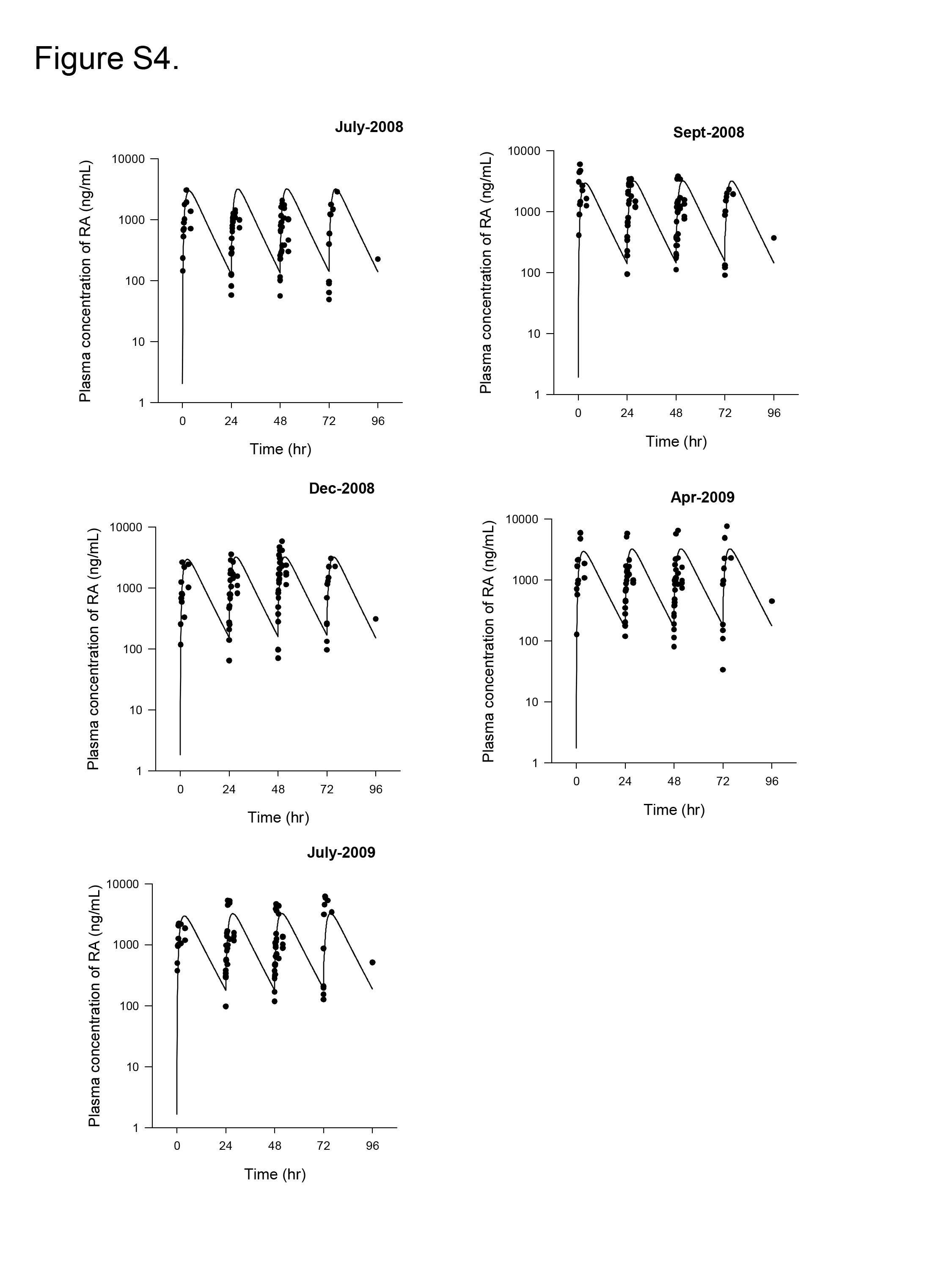

Supplement: Figure S4 — Plasma concentrations obtained after repeated oral dosing of juvenile male monkeys with MPH (NCTR data). Data represent model simulated (lines) and observed (circles) individual plasma concentrations of RA (•) after repeated oral dosing with 12.5 mg/kg MPH (n = 1–4 at each time point) across the study. Measurements of plasma RA concentrations at pre-dose (approximately within 30 min of dosing) were combined with those at 24 h from previous dose. MPH was administered twice a day, five days a week (Monday to Friday) and kinetic studies were performed from Monday to Thursday. On the day of blood collection, MPH was administered only once in the morning (solid lines). Dashed lines represent plasma concentration time courses of MPH and RA under repeated dosing schedules. (TIF) [file pone.0106101.s004.tif]

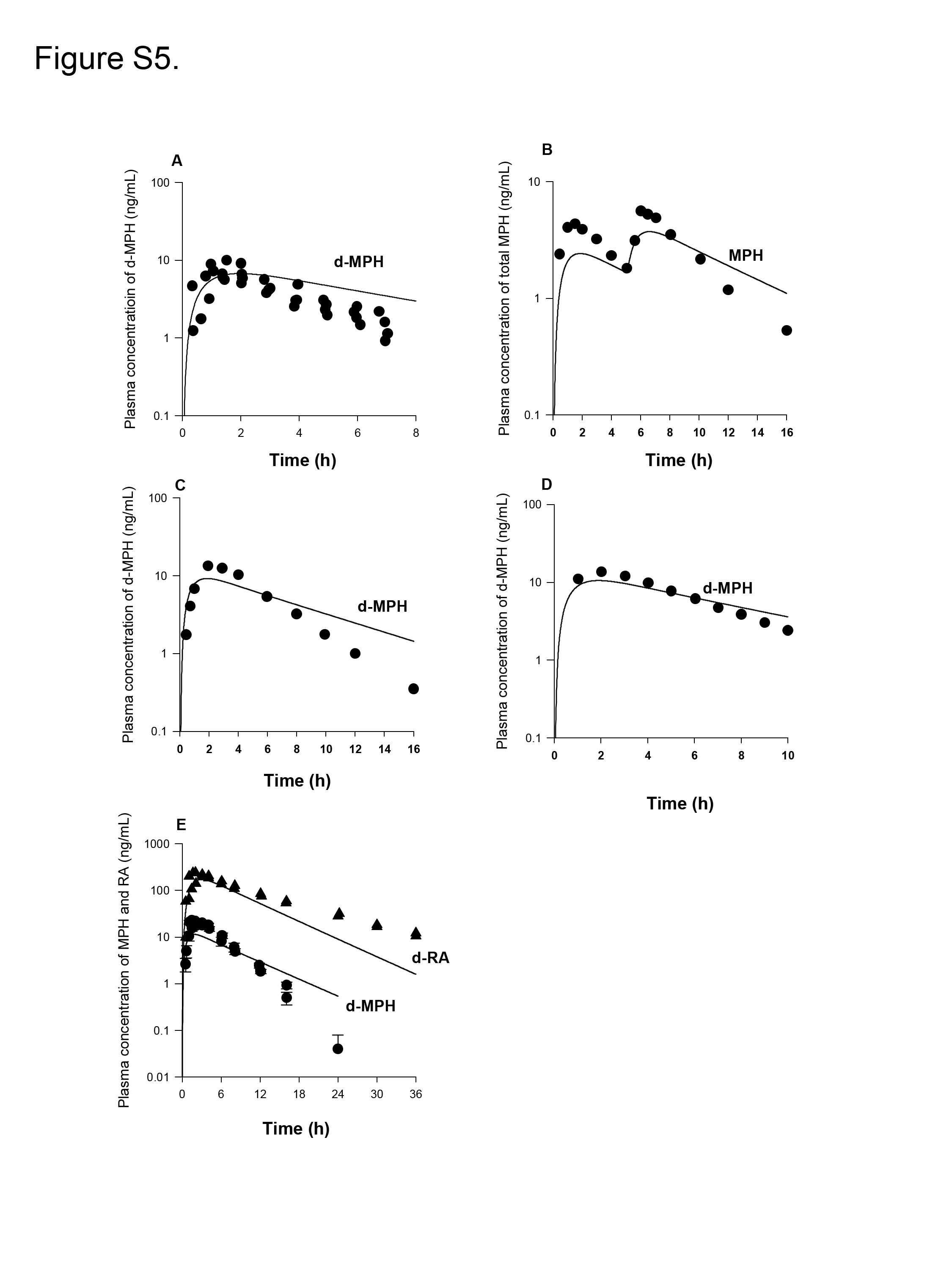

Supplement: Figure S5 — Plasma concentrations obtained after oral dosing of healthy adult humans with MPH. Panel A: Data represent model simulated (lines) and observed individual (circles) plasma concentrations of d-MPH after oral dosing with 20 mg MPH (n = 4) [40]; Panel B: Data represent model simulated (line) and observed (circles) plasma concentrations of MPH after two repeated dosing with 10 mg MPH, taken 5 h apart (n = 18) [43]; Panel C: Data represent model simulated (line) and observed (circles) plasma concentrations of d-MPH after oral dosing with 40 mg of MPH (n = 24) [39]; Panel D: Data as described for Panel A obtained after oral dosing with 40 mg MPH (n = 6) [42]; Panel E: Data represent model simulated (lines) and observed plasma concentrations of d-MPH (•) and d-RA(▴) after oral dosing with 20 mg d-MPH (n = 15) [41]. (TIF) [file pone.0106101.s005.tif]

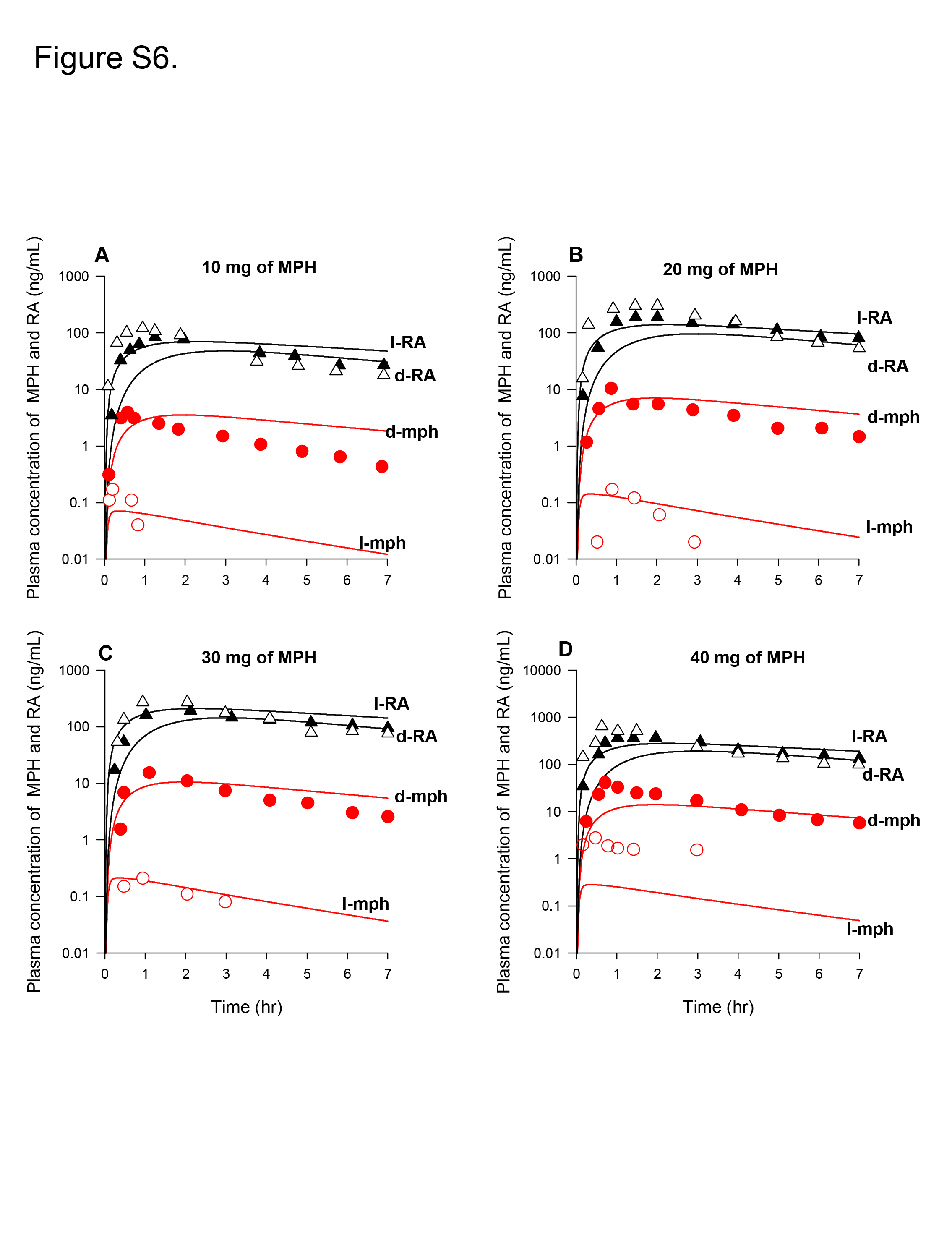

Supplement: Figure S6 — Plasma concentrations obtained after oral dosing of adult humans with MPH. Panel A: Data represent model simulated (lines) and observed plasma concentrations of d-MPH (•), l-MPH (○), d-RA (▴) and l-RA(Δ) after oral dosing with 10 mg MPH (n = 1) [38]; Panel B: Data as described for Panel A obtained after oral dosing with 20 mg MPH (n = 1) [38]; Panel C: Data as described for Panel A obtained after oral dosing with 30 mg MPH (n = 1) [38]; Panel D: Data as described for Panel A obtained after oral dosing with 40 mg. (TIF) [file pone.0106101.s006.tif]
