# Supplementary material for: Development of a Physiologically Based Model to Describe the Pharmacokinetics of Methylphenidate in Juvenile and Adult Humans and Nonhuman Primates
Source: PLoS One. 2014 Sep 3;9(9):e106101. doi: 10.1371/journal.pone.0106101 (PMC4153582; doi:10.1371/journal.pone.0106101)
Supplement: Table S1 — Immediate release MPH pharmacokinetic studies used for model calibration and evaluation for healthy adult male and female humans. (DOC) [file pone.0106101.s007.doc]

**Table S1.** Immediate release MPH pharmacokinetic studies used for model calibration and evaluation for healthy adult male and female

humans.

| **Number of Subjects**  **(age, years)** | **Route of Administration**  **Single Dose (mg)** | **Plasma**  **Measurements** | **Reference** |
| --- | --- | --- | --- |
| **Model calibration** |  |  |  |
| 13 men (18-30) | iv 10 | *d*- and *l*-MPH |  |
| 9 men (18-30) | iv 10 | urinary *d*- and *l*-RA |  |
| 12 men (22-30) and 12 women (21-42) | oral 0.3 mg/kg | *d*- and *l*-MPH |  |
| 19 men and women (23-40) | oral 0.3 mg/kg | *d-* and *l*-MPH |  |
| 21 men (18-45) | oral 40 | *d*- and *l*-MPH |  |
| 5 men (20-26) | oral 20 | MPH and RA |  |
| 8 men (20-40) | oral 20 | MPH and RA |  |
| 10 men (21-40) | oral 0.15 and 0.3 mg/kg | MPH and RA |  |
|  |  |  |  |
| **Model evaluation** |  |  |  |
| 49 adults (18-45) | oral 50 and 90 | *d*-MPH |  |
| 23 men and 5 women (19-68) | oral 30 repeated dose | *d*-MPH |  |
| 20 men (20-33) | oral 20 | MPH |  |
| 35 adults (18-41) | oral 5 repeated dose | MPH and RA |  |
| 9 men (18-30) | oral 40 | urinary *d*- and *l*-RA |  |
| 3 men | oral 20 | urinary RA |  |
| 6 men and women (18-55) | oral 40 | *d*-MPH |  |
| 24 men (18-50) | oral 40 | *d*-MPH |  |
| 3 men and 1 woman (28-42) | oral 20 | *d*-MPH |  |
| 1 adult | oral 10-40 | *d*- and *l*-MPH and  *d*- and *l*-RA |  |
| 18 men | oral 10 repeated dose | MPH |  |
| 9 men and 6 women (20-44) | oral 20 *d*-MPH | *d*-MPH and *d*-RA |  |
