# Supplementary material for: Development of a Physiologically Based Model to Describe the Pharmacokinetics of Methylphenidate in Juvenile and Adult Humans and Nonhuman Primates
Source: PLoS One. 2014 Sep 3;9(9):e106101. doi: 10.1371/journal.pone.0106101 (PMC4153582; doi:10.1371/journal.pone.0106101)
Supplement: Table S2 — Immediate release MPH pharmacokinetic studies used for model calibration and evaluation for male and female children with ADHD and ADD. (DOC) [file pone.0106101.s008.doc]

**Table S2.** Immediate release MPH pharmacokinetic studies used for model calibration and evaluation for male and female children with ADHD and ADD.

| **Number of Subjects**  **(age, years)** | **Route of Administration**  **Single Dose (mg)** | **Plasma**  **Measurements** | **Reference** |
| --- | --- | --- | --- |
| **Model calibration** |  |  |  |
| 5 boys (7-13) | oral 10-15 | peak serum RA |  |
| 5 boys (8-13) | oral 10 | *d*- and *l*-MPH |  |
| 9 boys (11.1±1.7) | oral 10 | *d*- and *l*-MPH |  |
|  |  |  |  |
| **Model evaluation** |  |  |  |
| 14 preschool (4-6) and 9 school-aged (7-8) boys and girls | oral 2.5-10 | *d*-MPH |  |
| 31 boys (9-12) | oral 5-20 | *d*-MPH |  |
| 14 boys (7-12.4) | oral 0.34 and 0.65 mg/kg | MPH |  |
| 14 boys and girls (7-12) | oral 5-15 repeated dose | MPH |  |
| 13 boys and 1 girl (6-12) | oral 10-40 repeated dose | MPH |  |
