# Supplementary material for: Development of a Physiologically Based Model to Describe the Pharmacokinetics of Methylphenidate in Juvenile and Adult Humans and Nonhuman Primates
Source: PLoS One. 2014 Sep 3;9(9):e106101. doi: 10.1371/journal.pone.0106101 (PMC4153582; doi:10.1371/journal.pone.0106101)
Supplement: Table S3 — Human equivalent dose (HED) calculations for MPH in boys either 6 or 15 years of age based on juvenile male rhesus monkey toxicity and pharmacokinetic studies with MPH. For juvenile monkeys, experimental daily AUC was calculated as total AUC over one week divided by 5 days and adjusted daily AUC used for HED calculations was calculated as total AUC over one week divided by 7 days; for boys, daily AUC was calculated as (total AUC over one week − total AUC from Money to Wednesday) divided by 4 days. (DOC) [file pone.0106101.s009.doc]

**Table S3.** Human equivalent dose (HED) calculations for MPH in boys either 6 or 15 years of age based on juvenile male rhesus monkey toxicity and pharmacokinetic studies with MPH. For juvenile monkeys, experimental daily AUC was calculated as total AUC over one week divided by 5 days and adjusted daily AUC used for HED calculations was calculated as total AUC over one week divided by 7 days; for boys, daily AUC was calculated as (total AUC over one week − total AUC from Money to Wednesday) divided by 4 days.

| **Male Juvenile Monkeys** | | | |  | **Boys** | | | | |
| --- | --- | --- | --- | --- | --- | --- | --- | --- | --- |
| **MPH Dose**  **(mg/kg)** | **Cmax**  **(ng/mL)** | **Daily AUC**  **(ng/mL*h per day)** | |  | **HED using Cmax** | |  | **HED using AUC** | |
|  |  |  |  |  | **6 year** | **15 year** |  | **6 year** | **15 year** |
|  |  | **Experimental** | **Adjusted** |  | **mg/kg (mg)** | **mg/kg (mg)** |  | **mg/kg (mg)** | **mg/kg (mg)** |
| 2.5 | 10.4 | 70.4 | 50.3 |  | 0.183 (3.9) | 0.261 (15.5) |  | 0.084 (1.8) | 0.114 (6.8) |
