# Supplementary material for: Development of a Physiologically Based Model to Describe the Pharmacokinetics of Methylphenidate in Juvenile and Adult Humans and Nonhuman Primates
Source: PLoS One. 2014 Sep 3;9(9):e106101. doi: 10.1371/journal.pone.0106101 (PMC4153582; doi:10.1371/journal.pone.0106101)
Supplement: Text S1 — Additional pharmacokinetic studies in humans. (DOC) [file pone.0106101.s010.doc]

**SUPPLEMENTARY DATA**

**Additional pharmacokinetic studies in humans**

Additional pharmacokinetic studies were used for adult human oral model evaluation. The first data sets were time courses of plasma *d-*MPH concentrations in adult humans following a single oral dose of MPH at 40mg (n=6) , 40mg (n=24) , 20mg (n=4) , and 10-40mg (n=1) , for which plasma concentrations for *l-*MPH, *d-*RA and *l-*RA were also determined. The second data set was time course of MPH plasma concentrations in adult men administered repeated doses of 10 mg MPH (n=18) . In addition, the calibrated model was evaluated against plasma *d-*MPH and *d*-RA concentrations in adults given 20 mg *d-*MPH (n=15) .
